# Proximity-based labeling reveals DNA damage–induced phosphorylation of fused in sarcoma (FUS) causes distinct changes in the FUS protein interactome

**DOI:** 10.1016/j.jbc.2022.102135

**Published:** 2022-06-14

**Authors:** Michelle A. Johnson, Thomas A. Nuckols, Paola Merino, Pritha Bagchi, Srijita Nandy, Jessica Root, Georgia Taylor, Nicholas T. Seyfried, Thomas Kukar

**Affiliations:** 1 Department of Pharmacology and Chemical Biology, Emory University, School of Medicine, Atlanta, Georgia, USA; 2 Center for Neurodegenerative Disease, Emory University, School of Medicine, Atlanta, Georgia, USA; 3 Emory Integrated Proteomics Core, Emory University, School of Medicine, Atlanta, Georgia, USA; 4 Department of Neurology, Emory University, School of Medicine, Atlanta, Georgia, USA; 5 Department of Biochemistry, Emory University, School of Medicine, Atlanta, Georgia, USA

**Keywords:** fused in sarcoma, DNA damage, phosphorylation, proteomics, translation, protein–protein interaction, protein translocation, frontotemporal dementia, amyotrophic lateral sclerosis, ALS, amyotrophic lateral sclerosis, APEX2, ascorbate peroxidase 2, Baf, bafilomycin, co-IP, coimmunoprecipitation, DNA-PK, DNA-dependent protein kinase, DPBS, Dulbecco’s PBS, DSB, double-strand DNA break, EGFP, enhanced GFP, FC, fold change, FTD, frontotemporal dementia, FTLD, frontotemporal lobar degeneration, FUS, fused in sarcoma, FUS P525L, FUS proline 525 to leucine mutant, FUS PM, phosphomimetic variant of FUS, FUS WT, wildtype FUS, GO, Gene Ontology, HEK293T, human embryonic kidney 293T cell line, IF, immunofluorescence, LFQ, label-free quantitation, MS, mass spectrometry, NMD, nonsense-mediated decay, PIC, protease inhibitor cocktail, PNS, postnuclear supernatant, PTM, post-translational modification, qPCR, quantitative PCR, RIPA, radioimmunoprecipitation assay, RT, room temperature, SAINT, Significance Analysis of INTeractome, SUnSET, SUrface SEnsing of Translation, TBS, Tris-buffered saline, TBST, TBS with Tween-20

## Abstract

Accumulation of cytoplasmic inclusions containing fused in sarcoma (FUS), an RNA/DNA-binding protein, is a common hallmark of frontotemporal lobar degeneration and amyotrophic lateral sclerosis neuropathology. We have previously shown that DNA damage can trigger the cytoplasmic accumulation of N-terminally phosphorylated FUS. However, the functional consequences of N-terminal FUS phosphorylation are unknown. To gain insight into this question, we utilized proximity-dependent biotin labeling *via* ascorbate peroxidase 2 aired with mass spectrometry to investigate whether N-terminal phosphorylation alters the FUS protein–protein interaction network (interactome), and subsequently, FUS function. We report the first analysis comparing the interactomes of three FUS variants: homeostatic wildtype FUS (FUS WT), phosphomimetic FUS (FUS PM; a proxy for N-terminally phosphorylated FUS), and the toxic FUS proline 525 to leucine mutant (FUS P525L) that causes juvenile amyotrophic lateral sclerosis. We found that the phosphomimetic FUS interactome is uniquely enriched for a group of cytoplasmic proteins that mediate mRNA metabolism and translation, as well as nuclear proteins involved in the spliceosome and DNA repair functions. Furthermore, we identified and validated the RNA-induced silencing complex RNA helicase MOV10 as a novel interacting partner of FUS. Finally, we provide functional evidence that N-terminally phosphorylated FUS may disrupt homeostatic translation and steady-state levels of specific mRNA transcripts. Taken together, these results highlight phosphorylation as a unique modulator of the interactome and function of FUS.

Frontotemporal lobar degeneration (FTLD) is a neurodegenerative disease characterized by atrophy of the frontal and temporal lobes. Frontotemporal dementia (FTD) is the clinical manifestation of FTLD (1). FTD is a heterogenous group of clinical disorders characterized by (a) alterations in behavior and personality and/or (b) impairments in language comprehension and communication (1, 2). Pathological and genetic similarities between FTD and another neurodegenerative disease, amyotrophic lateral sclerosis (ALS), suggest that FTD and ALS exist on a disease spectrum (3, 4, 5, 6). ALS is a progressive motor neuron disease characterized by degeneration of upper and lower motor neurons (3, 7). While ALS and FTLD cases typically vary in symptom presentation, a large subset of FTLD and ALS cases display intraneuronal cytoplasmic aggregates containing the fused in sarcoma (FUS) protein (8, 9, 10, 11). Specifically, ∼10% of FTLD cases and ∼5% of ALS cases exhibit FUS inclusions (1, 12, 13). These cases are termed FTLD-FUS and ALS-FUS, respectively (14, 15, 16, 17).

FUS is a widely expressed pleiotropic RNA/DNA-binding protein involved in gene transcription, DNA-repair pathways, mRNA splicing, mRNA transport, and stress granule assembly (15, 18, 19, 20, 21, 22, 23, 24, 25). Cellular dysfunction related to FUS is thought to be driven by novel gain of functions, including alterations in mRNA splicing, transcript expression, and the DNA damage response. Furthermore, the cytoplasmic accumulation of FUS is sufficient to promote cell death (26, 27, 28, 29, 30, 31). However, it remains unclear what triggers of FUS mislocalization are essential in eliciting FTLD–ALS disease pathogenesis.

Various factors may contribute to the development of cytoplasmic FUS inclusions. Genetic mutations in *FUS* typically cause ALS and are rarely associated with FTLD (8, 11, 32, 33). Thus, the proximal cause of FUS pathology in FTLD is unknown. One possibility is that FUS pathology is caused by exposure to an environmental toxin or dysregulated post-translational modifications (PTMs), such as phosphorylation or methylation (21, 34, 35, 36, 37, 38, 39, 40). Phosphorylation is a reversible PTM that regulates the function of numerous proteins in the cell (41). Abnormal or dysregulated protein phosphorylation is a common feature of many neurodegenerative disorders, including FTLD and ALS (42, 43). FUS can be phosphorylated at multiple N- and C-terminal residues (35, 44, 45, 46, 47). Our laboratory discovered that phosphorylation of 12 specific N-terminal residues in FUS by the DNA-dependent protein kinase (DNA-PK) causes the cytoplasmic accumulation of phosphorylated FUS (39, 46, 48). This cascade is triggered by double-strand DNA breaks (DSBs). Studies have found that FTLD and ALS exhibit markers of DNA damage (37, 46, 49). Given this, the cytoplasmic relocalization of FUS induced by N-terminal phosphorylation may contribute to pathology in a subset of FTLD and ALS cases. However, it remains unclear whether N-terminal phosphorylation alters FUS function. Therefore, we aimed to elucidate how the FUS protein interactome changed in response to phosphorylation at these 12 key N-terminal residues.

Chemically induced DSBs not only lead to robust phosphorylation of FUS but also induce multiple kinases and DNA repair cascades that may mask the specific effect of N-terminal FUS phosphorylation and make proteomic analysis challenging (50). To overcome this hurdle, we expressed a phosphomimetic variant of FUS (FUS PM) that mimics the cytoplasmic localization of FUS caused by DSBs (46, 48). We engineered synthetic genes that fused ascorbate peroxidase 2 (APEX2) to human wild-type FUS (FUS WT), FUS PM, or the ALS-linked mutant P525L (FUS P525L [FUS proline 525 to leucine mutant]) to enable proximity-dependent biotinylation of potential protein-binding partners (51). We then performed label-free mass spectrometry (MS) on biotinylated proteins to determine whether N-terminal phosphorylation alters the protein-binding partners of FUS (51). Differential expression analysis revealed that the FUS PM interactome compared with FUS WT was enriched for cytoplasmic proteins involved in “mRNA catabolic process,” “translation initiation,” and “stress granule assembly.” In contrast, the FUS PM interactome compared with FUS P525L was enriched for nuclear proteins involved in functions, such as “spliceosome,” “ribonucleoprotein complex biogenesis,” and “covalent chromatin modification.” We found that cells expressing FUS PM exhibited functional alterations in the steady-state levels of certain mRNAs and global translation. Taken together, these data suggest that phosphorylation results in a novel FUS interactome that exists between the pathogenic FUS P525L ALS-linked mutation and the homeostatic functions of FUS WT. Our analysis is the first comprehensive study how a disease-relevant PTM in FUS may shift its protein interactome toward a disease state. Findings from these studies will inform how phosphorylation of FUS and the ALS-linked FUS mutation P525L contribute to neurodegeneration.

## Results

### APEX2-tagged FUS PM recapitulates p-FUS localization phenotype

**Figure 1:**
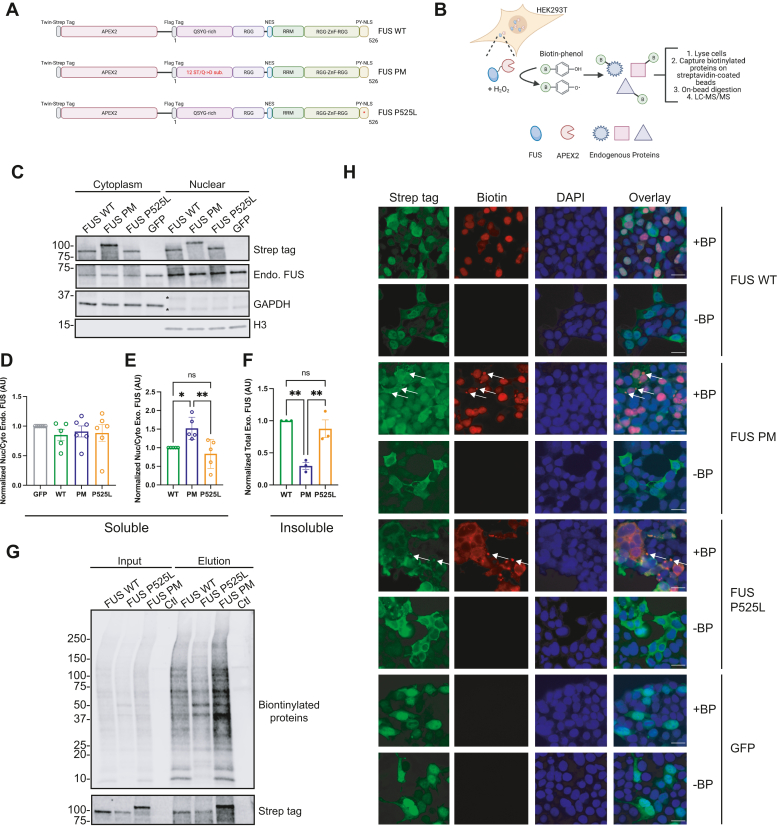
**APEX2-FUS variants generate unique biotinylation patterns based on subcellular localization.** Graphical representation of the three APEX2-FUS fusion constructs used in this work. Each construct contains a Twin-Strep tag to facilitate detection in downstream applications, APEX2, a linker sequence, a FLAG tag, and a variant of human full-length FUS. The three FUS variants are wildtype FUS (FUS WT), phosphomimetic FUS (FUS PM) where either serine or threonine at the 12 DNA-PK consensus sites (S/T-Q) was mutated to aspartate (D), and pathogenic P525L mutant FUS (FUS P525L). *B*, schematic workflow of APEX2 induced biotin proximity labeling coupled with liquid chromatography and tandem mass spectrometry (LC–MS/MS) to define the FUS protein interactome. *A* and *B* were created with BioRender.com. *C*, HEK293T cells expressing the three APEX2-FUS fusion constructs were fractionated for cytoplasmic and nuclear fractions. GAPDH and H3 were used as markers for cytoplasmic and nuclear fractions, respectively. ∗Nonspecific bands from GAPDH antibody. *D*, quantification of (*C*) for the nuclear (nuc):cytoplasmic (cyto) ratio of detergent-soluble Strep-tagged APEX2 fusion protein ratio and normalized to total protein. *E*, quantification of (*C*) for the nuc:cyto ratio of detergent-soluble endogenous FUS normalized to total protein. *F*, quantification of Strep-tagged APEX2 fusion proteins found in the detergent-insoluble fraction and normalized to total protein (immunoblot not shown). *G*, enrichment of biotinylated proteins from HEK293T cells expressing APEX2-FUS constructs and treated with biotin–phenol and H_2_O_2_. Input is 1% of sample loaded onto magnetic beads coated with streptavidin; elute is 10% of sample eluted off beads. Samples are FUS WT, FUS P525L, FUS PM, and nontransfected control (CTL). Input and elution were analyzed for biotinylated proteins (streptavidin) and Twin-Strep tag (Strep tag). *H*, immunostaining of HEK293T cells expressing the three APEX2-FUS fusion constructs or Strep-GFP that have been given biotin–phenol (+BP) and H_2_O_2_ for Twin-Strep tag (fusion protein) and streptavidin (biotin). Cytoplasmic puncta observed in FUS PM and P525L samples treated with BP (*white arrows*). The scale bar represents 20 μm. APEX2, ascorbate peroxidase 2; AU, arbitrary unit; DNA-PK, DNA-dependent protein kinase; FUS, fused in sarcoma; HEK293T, human embryonic kidney 293T cell line.

FUS dysfunction is a hallmark of FTD and ALS disease pathogenesis (6, 9, 52). However, many fundamental aspects of FUS regulation are unknown. For example, it remains unclear how phosphorylation of FUS, or the presence of ALS-associated mutations, alters the function of FUS and associated pathways. To gain insight into these questions, we set out to define the protein-binding network, or interactome, of FUS by performing proximity labeling mediated by APEX2 (51). We fused APEX2 to the N terminus of three FUS protein variants *via* a (GGGS)^3^-FLAG tag linker to generate three Twin-Strep-tagged constructs: (1) FUS WT, (2) FUS PM, and (3) the ALS-linked FUS P525L (Fig. 1, *A* and *B*). FUS PM was generated by substituting the 12 serine/threonine residues that are phosphorylated by DNA-PK following DSB with the negatively charged amino acid aspartate (46, 48). FUS P525L was generated by substituting leucine for proline at position 525. The FUS P525L mutation, which causes a severe form of juvenile ALS, robustly increases cytoplasmic localization of FUS and alters the transcriptome, proteome, and the spliceosome in multiple model systems (18, 53, 54, 55, 56). Therefore, the APEX2-FUS P525L mutant (1) served as a positive control for FUS cytoplasmic localization, (2) provided insight into the pathogenic nature of ALS-linked mutations, and (3) was a useful comparison to determine if FUS PM resembles a known pathogenic phenotype (44, 57).

We first asked if fusion of APEX2 maintained the expected subcellular localization of the FUS variants. We expressed the three APEX2 fusion constructs in human embryonic kidney 293T (HEK293T) cells and biochemically fractionated cells into a soluble cytoplasmic and nuclear fraction (Fig. 1*C*). Endogenous FUS protein was enriched in the nuclear fraction, and the ratio of cytoplasmic/nuclear FUS was unchanged regardless of APEX2-fusion protein expression, suggesting expression of our APEX2 fusion constructs did not disrupt endogenous FUS expression (Fig. 1, *C* and *D*). Previously, we reported that the cytoplasmic localization of phosphorylated FUS induced by DSB can be mimicked by phosphomimetic substitution of the 12 consensus DNA-PK phosphorylation sites (serine/threonine_glutamine [S/T_Q]) with aspartate (D) (46). As anticipated, a larger proportion of APEX2-FUS PM was found in the cytoplasm compared with the nucleus *via* immunoblot (Fig. 1*E*). FUS-ALS mutations such as P525L typically induce an accumulation of FUS into insoluble cytoplasmic inclusions (9, 13). Accordingly, we examined the insoluble protein fraction and found that APEX2-FUS WT and APEX2-P525L FUS were both significantly increased in the insoluble fraction compared with APEX2-FUS PM (Fig. 1*F*). This suggests that a significant fraction of APEX2-FUS WT and APEX2-FUS P252L is detergent insoluble (Fig. 1*F*). It should be noted that increased deposition of insoluble FUS is correlated with cellular toxicity (58). Furthermore, insoluble APEX2-FUS WT or APEX2-P525L protein could localize to either the nucleus or the cytoplasm as insoluble aggregates of P525L have been reported in both the nucleus and the cytoplasm (59, 60). Therefore, we next utilized immunofluorescent staining to determine the subcellular localization of the APEX2 fusion proteins without relying on detergent-based biochemical fractionation. In line with Western blot analysis, APEX2-FUS WT was found in the cytoplasm and nucleus. In contrast, both APEX2-FUS PM and APEX2-FUS P525L showed a more pronounced cytoplasmic localization and occasional formation of cytoplasmic puncta (Fig. 1*H*). Taken together, our data demonstrate that the APEX2 fusion FUS variants localize to expected cellular compartments.

### APEX2-FUS variants exhibit unique biotinylation patterns

To further validate the proximity ligation system, we confirmed that the APEX2 fusion proteins are active and can biotinylate endogenous proteins. APEX2 requires the addition of biotin–phenol and H_2_O_2_ to catalyze the biotinylation of proximal endogenous proteins (Fig. 1, *B* and *G*). When we treated HEK293T cells expressing the APEX2-FUS variants with biotin–phenol and H_2_O_2_, we observed robust and variant-specific biotin labeling of endogenous proteins as detected by immunofluorescence (IF) with streptavidin (Fig. 1*H*). In contrast, we did not observe biotin labeling in cells that were not treated with biotin–phenol or H_2_O_2_ (Fig. S1). While APEX2-FUS WT exhibited a mixed nuclear and cytoplasmic localization when immunostained for the Twin-Strep tag (Fig. 1*H*), it induced a primarily nuclear biotinylation pattern as determined by colocalization with streptavidin (biotin) and 4′,6-diamidino-2-phenylindole IF. APEX2-FUS PM exhibited a diffuse cytoplasmic localization pattern with biotinylated proteins primarily labeled in the nucleus with interspersed cytoplasmic puncta (*white arrows*). APEX2-FUS P525L was localized primarily to the cytoplasm and induced biotinylation in the cytoplasm along with cytoplasmic puncta (*white arrows*). Negative control cells expressing a Strep-tagged GFP show no biotinylation following biotin–phenol and H_2_O_2_ addition. These results demonstrate that APEX2-FUS variants exhibit unique and specific patterns of biotinylation.

Next, to identity the specific binding partners of the various APEX2-FUS proteins, we transfected HEK293T cells with APEX2-FUS WT, APEX2-FUS PM, or APEX2-FUS P525L for 24 h. Untransfected HEK293T cells were grown in parallel for 24 h and served as a control group. All biological groups contained four technical replicates. We incubated each experimental group of cells with biotin–phenol for 30 min followed by H_2_O_2_ for 1 min to induce biotinylation of proximal endogenous proteins. The reaction was quenched, and lysates were collected (Fig. 1*G*). While control cells did not receive biotin–phenol, they did receive H_2_O_2_ and underwent all downstream processing. Biotinylated proteins were enriched from the cell lysates using streptavidin affinity purification. Western blot analysis of ∼10% of the volume of streptavidin beads confirm enrichment of biotinylated proteins and revealed that each APEX2 FUS variant showed a distinct biotinylation pattern (Fig. 1*G*). The remaining affinity-purified biotinylated proteins were used for unbiased proteomic analysis.

### APEX2-induced biotinylation identifies novel binding partners of FUS variants

To identify novel FUS interacting proteins across WT and mutant FUS proteins, we performed MS-based proteomics using label-free quantitation (LFQ). A total of 4954 unique proteins were identified and quantified across all 16 samples (four technical replicates across four conditions). Significance Analysis of INTeractome (SAINT) analysis was performed to eliminate contaminating proteins and determine confidence scores of putative interactions (prey) for each APEX2-FUS (bait) (61). Prey with spurious interactions across all four conditions (*sensu* SAINT analysis; probability <0.95) was eliminated from further analysis. Finally, the mean intensity of the control samples for each identified protein was subtracted from the sample intensity value for the remaining prey proteins, leaving 3349 proteins classified as putative interacting proteins in at least one sample (Table S1).

**Figure 2:**
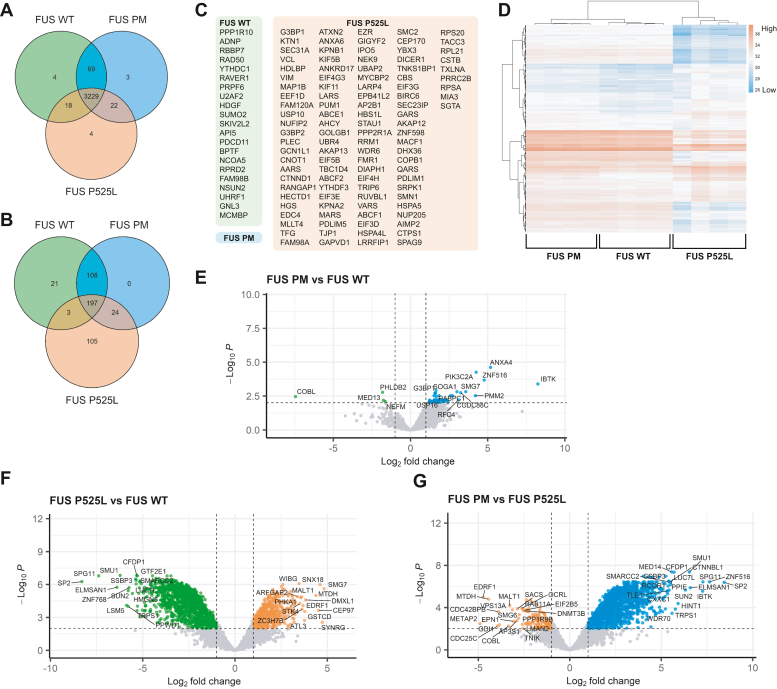
**FUS WT, FUS PM, and FUS P525L have unique protein interactomes.***A*, Venn diagram showing unique and overlapping proteins of all proteins identified in the APEX2-FUS WT (*green circle*), APEX2-FUS PM (*blue circle*), and APEX2-FUS P525L (*orange circle*) proteomes. *B*, Venn diagram showing overlap of the top 10% of the most enriched proteins for the three different APEX2-FUS variant proteomes. *C*, the top 10% of the most enriched proteins that were exclusively identified for the three different APEX2-FUS variants. *Boxes* are shaded to correspond to each FUS variant as in *A* and *B*. *D*, hierarchical clustering of APEX2 FUS samples based on the intensity profiles of the top 10% of protein hits. Missing values are colored *gray*. *E*, volcano plot of statistically significant differentially enriched (DE) proteins identified comparing FUS PM *versus* FUS WT. Proteins that pass cutoff (*p* < 0.01, logFC > |1|) are colored *blue* (enriched in FUS PM/FUS WT) or *green* (enriched in FUS WT/FUS PM). *F*, volcano plot of statistically significant DE proteins identified comparing FUS P525L and FUS WT. Proteins that pass cutoff (*p* < 0.01, logFC > |1|) are colored *orange* (enriched in FUS P525L/FUS WT) or *green* (enriched in FUS WT/FUS P525L). *G*, volcano plot of statistically significant DE proteins identified comparing FUS PM *versus* FUS P525L. Proteins that pass cutoff (*p* < 0.01, logFC > |1|) are colored *blue* (enriched in FUS PM/FUS P525L) or *orange* (enriched in FUS PM/FUS P525L). Volcano plots in *E*–*G* are based on differential expression of all identified protein hits. APEX2, ascorbate peroxidase 2; FUS, fused in sarcoma; FUS P525L, FUS proline 525 to leucine mutant; FUS PM, phosphomimetic variant of FUS.

Of the 3349 proteins that met our filtering criteria, 3229 (96.4%) were present in all three groups (Fig. 2*A*). However, visualization of the degree of enrichment between APEX2-FUS variants using unsupervised hierarchical clustering, heatmaps, and principal component analysis revealed that the magnitude of enrichment for each protein differed between variants (Figs. 2*D* and S2). Given this, we reasoned that the proteins most enriched in each APEX2-FUS variant may be unique; therefore, we compared the most abundant proteins for each group, using 10% as an arbitrary cutoff to exemplify the differences in enrichment between the groups (Fig. 2*B*). We identified a total of 458 proteins in the top 10% of biotinylated proteins across the three variants. Unlike the full dataset of proteins (Fig. 2*A*), only 197 proteins (43.0%) were shared between the three groups suggesting that each variant preferentially bound a distinct subset of proteins (Fig. 2*B*). In addition, we identified 21 proteins uniquely enriched in the top 10% of biotinylated proteins for APEX2-FUS WT and 105 proteins uniquely enriched for APEX2-FUS P525L (Fig. 2, *B* and *C*). In contrast, APEX2-FUS PM shared 108 proteins with APEX2-FUS WT and 24 proteins with APEX2-FUS P525L. These data suggest that FUS PM may exist in a functional state between FUS WT and FUS P525L, allowing it to interact with proteins that preferentially bind either homeostatic FUS WT or toxic FUS P525L.

Next, we compared the relative abundance of all identified proximity biotinylated proteins between the FUS PM and FUS WT variants (Fig. 2*E*), the FUS P525L and FUS WT variants (Fig. 2*F*), and the FUS PM and FUS P525L variants (Fig. 2*G*). For each comparison, we utilized a stringent cutoff of *p* < 0.01 and a log fold change (FC) >|1| to produce a dataset of significantly enriched proteins for each variant. About 53 proteins (1.6% of total identified proteins) differed between FUS WT and FUS PM, 1325 proteins (39.6% of total identified proteins) differed between FUS PM and FUS P525L, and 1600 proteins (47.8% of total identified proteins) differed between FUS WT and FUS P525L (Table S2). We then used MetaScape to compare the ontologies (*e.g.*, Gene Ontology [GO], Kyoto Encylopedia of Genes and Genomes processes, reactome gene sets, canonical pathways, and CORUM complexes) of differentially expressed proteins to gain insight into biological processes or functional categories that may be altered by each FUS variant (62) (Table 1). Of the 53 proteins differentially expressed in APEX2-FUS PM over APEX2-FUS WT, the top ontology categories are “mRNA catabolic process,” “translational assembly,” “stress granule assembly,” and “clathrin-mediated endocytosis” (Table 1). These functional categories are localized to the cytoplasm suggesting FUS PM participates in more cytoplasmic pathways compared with FUS WT.

**Table 1:** Comparison of GO and reactome pathways enriched in APEX2-FUS WT, PM, and P525L proteomes

Comparison	Pathway identifier	Description	−Log10 (*q* value)
FUS PM *versus* FUS WT			
Up in FUS PM	GO: 0006402	mRNA catabolic process	6.84
Up in FUS PM	GO: 0006413	Translational assembly	3.90
Up in FUS PM	GO: 0034063	Stress granule assembly	2.29
Up in FUS PM	R-HSA-8856828	Clathrin-mediated endocytosis	2.16
FUS PM *versus* FUS P525L			
Up in FUS PM	CORUM: 351	Spliceosome	96.90
Up in FUS PM	GO: 0022613	Ribonucleoprotein complex biogenesis	90.78
Up in FUS PM	GO: 0016569	Covalent chromatin modification	87.14
Up in FUS PM	GO: 0006281	DNA repair	80.15
Down in FUS PM	R-HSA-199991	Membrane trafficking	12.87
Down in FUS PM	GO: 0048193	Golgi vesicle transport	4.84
Down in FUS PM	GO: 0120031	Plasma membrane–bounded cell projection assembly	3.99
Down in FUS PM	GO: 016482	Cytosolic transport	3.99
FUS P525L *versus* FUS PM			
Down in FUS P525L	CORUM: 351	Spliceosome	96.72
Down in FUS P525L	GO: 0022613	Ribonucleoprotein complex biogenesis	90.74
Down in FUS P525L	GO: 0016569	Covalent chromatin modification	88.25
Down in FUS P525L	GO: 0006281	DNA repair	77.70
Down in FUS P525L	GO: 0050684	Regulation of mRNA processing	74.98
Up in FUS P525L	R-HSA-199991	Membrane trafficking	39.20
Up in FUS P525L	GO: 0006412	Translation	37.71
Up in FUS P525L	GO: 0048193	Golgi vesicle transport	17.97
Up in FUS P525L	GO: 0030029	Actin filament–based process	17.72

Table of statistically enriched GO and reactome pathways generated using MetaScape, a web-based platform designed to provide users a comprehensive annotation of provided gene list.

We also identified a subset of novel binding partners for the variants of FUS in our datasets. For example, four proteins were significantly enriched in FUS WT over FUS PM. These proteins were COBL (Cordon-bleu WH2 repeat protein), PHLDB2 (Pleckstrin homology–like domain family B member 2), MED13 (Mediator of RNA polymerase II transcription subunit 13), and NEFM (Neurofilament medium chain). Furthermore, the top four enriched proteins for FUS PM compared with FUS WT were IBTK (Inhibitor of Bruton tyrosine kinase), PIK3C2A (Phosphatidylinositol-4-phosphate 3-kinase catalytic subunit type 2 alpha), ZNF516 (Zinc finger protein 516), and ANXA4 (Annexin A4). To our knowledge, these are all novel putative binding partners for FUS.

About 1325 proteins were differentially enriched between APEX2-FUS PM and APEX2-FUS P525L (Fig. 2*G*). Of these proteins, ontology analysis revealed FUS PM enriched for proteins associated with functions in the nucleus including “spliceosome,” “ribonucleoprotein complex biogenesis,” “covalent chromatin modification,” and “DNA repair.” APEX2-FUS P525L enriched for pathways that occur in the cytoplasm including “membrane trafficking,” “Golgi vesicle transport,” “plasma membrane–bounded cell projection,” and “cytosolic transport” (Table 1). Finally, we identified 1600 proteins differentially enriched between APEX2-FUS WT and APEX2-FUS P525L (Fig. 2*F*). Of these proteins, ontology analysis revealed FUS WT enriched for proteins associated with the nuclear functions of “spliceosome,” “ribonucleoprotein complex biogenesis,” “covalent chromatin modification,” and “DNA repair,” whereas FUS P525L enriched for proteins associated with the cytoplasmic functions of “membrane trafficking,” “translation,” “Golgi vesicle transport,” and “actin filament–based process” (Table 1).

**Figure 3:**
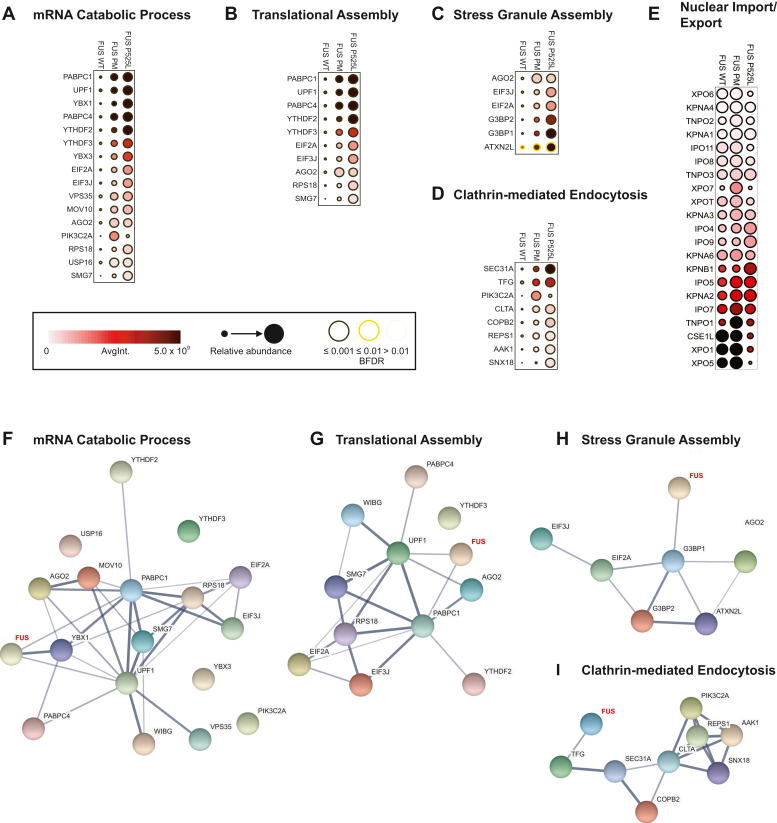
**Novel interaction partners of FUS variants identified through visualization of top protein hits in Gene Ontology (GO) and reactome pathways.***A*–*E*, dot plots generated using ProHits-viz graphically represent the relative abundance of proteins enriched in APEX2-FUS WT, PM, or P525L proteomes involved in (*A*) mRNA catabolic process, (*B*) translational assembly, (*C*) stress granule assembly, (*D*) clathrin-mediated endocytosis, and (*E*) nuclear import/export. *F*–*I*, protein interaction network for (*F*) mRNA catabolic process, (*G*) translational assembly, (*H*) stress granule assembly, and (*I*) clathrin-mediated endocytosis generated using STRING (version 11). Thickness of line between proteins indicates the strength of the empirical support for the interaction. FUS (in *red*) was added to each network to demonstrate known binding partners. APEX2, ascorbate peroxidase 2; FUS, fused in sarcoma; PM, phosphomimetic.

Next, we constructed dot plots to clearly visualize the intensity and confidence of the protein interaction across each APEX2-FUS variant using the ProHits-viz software suite (63). A summary of dot plots for all identified ontology categories can be found as supporting information (Fig. S3). We compared the binding partners identified in the top four significantly enriched ontology categories for FUS PM *versus* FUS WT (GO or reactome) (Fig. 3, *A–D*). The relative abundance of the target proteins for FUS WT, FUS PM, and FUS P525L variants tended to occur as low, medium, and high, respectively. This observation complements the original observation from the Venn diagram and the hierarchical cluster that FUS PM may exist in a functional state between FUS WT and FUS P525L function.

Because the function of FUS depends on shuttling between the nucleus and cytoplasm, we compared the abundance of proteins involved in nuclear import and export in the APEX2-FUS proteomes. We identified a total of 21 nuclear import or export receptor proteins shared across the APEX2-FUS WT, PM, and P525L proteomes (Fig. 3*E*). The relative abundance of most nuclear import and export receptors was similar between FUS WT and PM. However, the levels of exportin-7 (XPO7) and transportin-1 (TNPO1) were increased in the APEX2-FUS PM proteome compared with FUS WT and P525L. In contrast, the APEX2-FUS P525L proteome contained lower levels of multiple nuclear import and export proteins compared with FUS WT or PM, including exportin-6 (XPO6), transportin-2 (TNPO2), importin-11 (IPO11), importin-8 (IPO8), exportin-T (XPOT), karyopherin subunit alpha 6 (KPNA6), TNPO1, chromosome segregation 1 like/exportin-2 (CSE1L/XPO2), and exportin-5 (XPO5). Intriguingly, importin-4 (IPO4), importin-9 (IPO9), and importin subunit beta-1 (IMB1; KPNB1) were more abundant in the APEX2-FUS P525L proteome. These data demonstrate that APEX2-mediated proximity labeling is a useful method to broadly identify nuclear import and export receptors, which are often difficult to coimmunoprecipitate and do not always occur in other published FUS proteomes that use different methods (Table S3) (20, 64). Furthermore, these data suggest that PTMs and disease-associated mutations have complex effects on the FUS interactome.

Given that the top GO terms were generated from sets of enriched proteins, we wanted to visualize the known interactions between FUS and the target proteins in each gene set. We utilized the STRING database (version 11) to create an interaction network from each functional term (65) (Fig. 3, *F*–*I*). The STRING algorithm is built from a curated list of known protein interactions to estimate how likely the interaction is true given the available evidence (termed confidence). The confidence for each interaction is shown by the thickness of the line between each protein. In these networks, we observed with high confidence that FUS interacts with a subset of proteins in each network. Even so, there are few reports from previous studies indicating that FUS directly interacts with most of the proteins in each gene set. This may indicate that FUS WT interacts with more proteins in each interaction network than previously reported. Furthermore, these data suggests that N-terminal phosphorylation shifts the interaction landscape of FUS, allowing it to interact with more proteins central to these functional categories. As follows, we selecteda subset of proteins (both previously identified as direct interactions and novel interactions) from the gene sets to validate using traditional biochemical approaches (coimmunoprecipitation [co-IP] and IF): G3BP1, UPF1, MOV10, eIF2α, and PABPC1 (PABP1).

### Biochemical validation of FUS variant–binding partners reveals novel interactions between FUS variants and APEX2 hits

**Figure 4:**
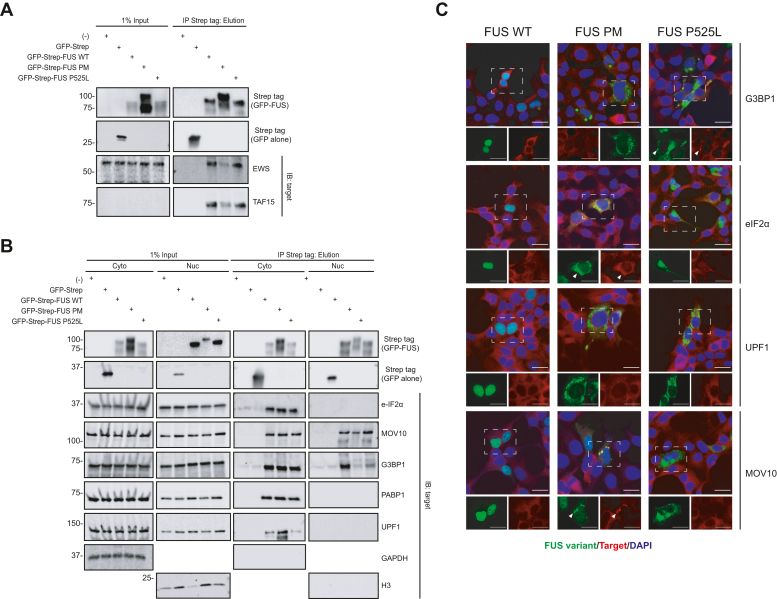
**Verification of the interaction between select targets and FUS variants.***A*, immunoprecipitation (IP) of Strep tag-FUS variants was performed from whole-cell lysate of HEK293T cells expressing GFP-Strep-tagged FUS WT, FUS PM, or FUS P525L (GFP-Strep-FUS WT, GFP-Strep-FUS PM, GFP-Strep-FUS PM, and GFP-Strep-FUS P525L, respectively). Cell lysate (input) and elution from IP was immunoblotted (IB) for listed targets. Cell lysate from cells either untransfected (−) or expressing GFP tagged with Strep alone (GFP-Strep) was used as a control. *B*, IP of GFP-Strep tag-FUS variants was performed from cell lysate that had been fractionated into cytoplasm (Cyto) and nuclear (Nuc) fractions. Fractionated cell lysate was from HEK293T cells expressing GFP-Strep-FUS WT, GFP-Strep-FUS PM, GFP-Strep-FUS PM, or GFP-Strep-FUS P525L. Enriched Cyto or Nuc fractions were IB for listed targets. Fractioned cell lysate from cells either untransfected (−) or expressing GFP-Strep was used as a control. *C*, immunofluorescence (IF) images of localization patterns of FUS WT, FUS PM, and FUS P525L and select targets (G3BP1, eIF2a, UPF1, and MOV10). Colocalization of targets with FUS punctate is highlighted by *white arrowhead*. FUS variants are labeled in *green*, targets are in *red*, and DAPI is in *blue*. The scale bars represent 20 μm. DAPI, 4′,6-diamidino-2-phenylindole; FUS, fused in sarcoma; FUS P525L, FUS proline 525 to leucine mutant; FUS PM, phosphomimetic variant of FUS; HEK293T, human embryonic kidney 293T cell line.

We evaluated whether the FUS variants coimmunoprecipitated with the following selected endogenous targets: G3BP1, UPF1, MOV10, eIF2α, and PABPC1 (PABP1) (Fig. 4*A*). HEK293T cells were transfected with N-terminally GFP-Twin-Strep-tagged FUS WT, FUS PM, or FUS P525L, which allowed fluorescent visualization following transfection. Next, we generated a whole-cell lysate and enriched for the Strep-tagged FUS variants using Strep-TactinXT magnetic beads (IP) and immunoblotted for potential endogenous binding partners (Fig. 4*A*). First, we verified that EWS and TAF15, members of the FET protein family, were pulled down in our assay conditions as previously reported (Fig. 4*A*) (dot plot for EWS and TAF15 in Fig. S3) (66). Importantly, we did not detect any enrichment of EWS or TAF15 in the two negative control conditions: (1) no transfection, beads alone (−) or (2) transfection with GFP-Strep. Next, we performed a second round of transfections, isolated cytoplasmic and nuclear fractions, repeated the IP, and analyzed bead elutions *via* Western blot. We find that FUS WT and FUS P525L co-IP with UPF1, PABP1, G3BP1, and eIF2α as previously reported (Fig. 4*B*) (67, 68, 69, 70). Finally, we confirmed the interaction of MOV10 to our three FUS variants, validating this novel FUS interaction (Fig. 4*B*). This is the first report that FUS PM interacts with MOV10 or any of the tested proteins.

Given that the three FUS variants are enriched in different cellular compartments (Fig. 1*H*), we performed immunofluorescent staining for a subset of the top proteins to determine the spatial localization of the binding partners with the FUS variants (Fig. 4*C*; PABP1, EWS, and TAF15 not shown). We expressed GFP-Twin-Strep-tagged FUS WT, FUS PM, or FUS P525L in HEK293T and then costained for the endogenous target proteins. As expected, FUS WT was enriched in the nuclear compartment, whereas FUS PM and FUS P525L were enriched in the cytoplasm. The endogenous target proteins localized to cytoplasm. Given this, we saw spatial overlap of the endogenous target proteins MOV10, G3BP1, UPF1, MOV10, and eIF2α with FUS PM and FUS P525L. For G3BP1 and MOV10, this overlap, at times, occurred in large puncta with FUS P525L and FUS PM, respectively (Fig. 4*C*, *white arrow*). Thus, biochemical validation with IP and IF robustly replicates a subset of protein interaction partners of FUS identified in our APEX2-generated proteomic dataset.

### The steady-state level of ATF3 transcripts is increased, whereas global protein translation is enhanced in the presence of FUS PM

Following validation of protein targets identified by APEX2, we set out to test whether the functional pathways suggested by our enrichment analysis were altered by the expression of a given FUS variant. We utilized four N-terminally GFP/Twin-Strep-tagged FUS constructs: (1) WT human FUS (WT), (2) human FUS where the 12 serine/threonine residues phosphorylated by DNA-PK are substituted with alanine (Ala sub), (3) human FUS where the 12 serines/threonines phosphorylated by DNA-PK have been substituted with the negatively charged aspartate (PM), (4) human FUS truncated before exon 15 (FUSΔ15), which lacks the C-terminal PY-NLS. We utilized the FUSΔ15 truncation mutant as a proxy for the FUS P525L mutation because deletion of the amino acids encoded by exon 15 disrupts binding of FUS to TNPO1 similarly to P525L and increases cytoplasmic localization (27, 30). We transfected pcDNA3.1 encoding GFP-Strep as a control.

**Figure 5:**
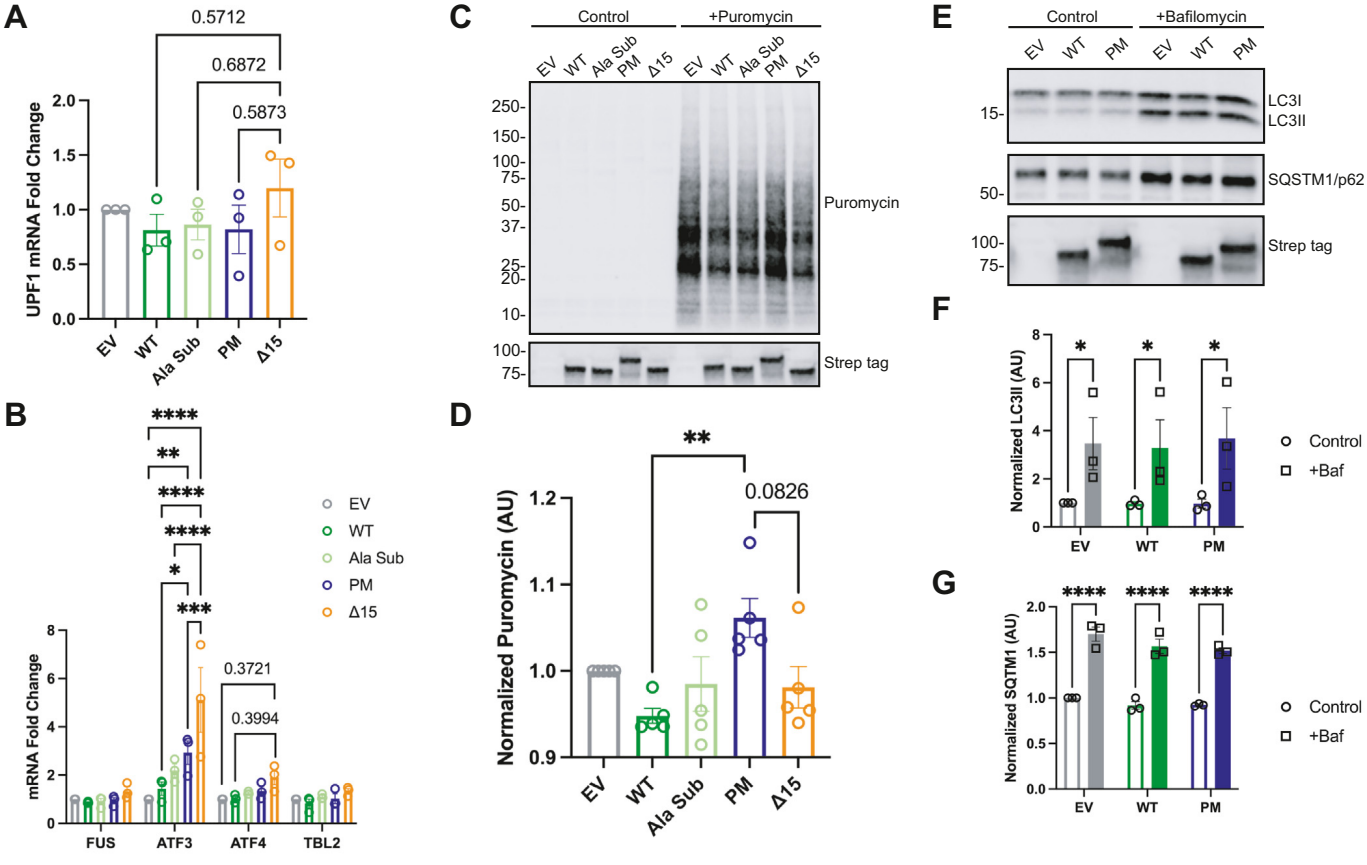
**FUS variants differentially alter nonsense-mediated mRNA decay and translation.***A*, UPF1 mRNA levels were quantified by qPCR using the ΔΔ cycle threshold (ΔΔCT) method. Fold change was calculated against the empty vector control (EV). *B*, levels of various stress-related targets of mRNA decay were quantified by qPCR using the ΔΔCT method, and then, the fold change was calculated against the empty vector control (EV). *C*, representative immunoblot of SUnSET assay measuring the incorporation of puromycin into growing polypeptide chains during translation. Control cells received Hepes buffer without puromycin for 30 min. *D*, quantification of immunoblot in (*C*). Error bars indicates mean ± SEM (n = 5). Statistical significance was calculated by one-way ANOVA. *E*, representative immunoblot for markers of autophagosome flux, LC3I/II and SQSTM1/p62. *F* and *G*, quantification of LC3II and SQSTM1 immunoblot in (*E*). Error bars indicate mean ± SEM (n = 3). Statistical significance was calculated by two-way ANOVA. FUS, fused in sarcoma; qPCR, quantitative PCR; SUnSET, SUrface SEnsing of Translation.

We specifically focused on the pathways enhanced by FUS PM expression. The highest enriched ontology category for FUS PM over FUS WT was “mRNA catabolic process,” defined as the reactions and pathways associated with the breakdown of mRNA (Table 1). As an RNA/DNA-binding protein, FUS expression has been shown to regulate ∼700 mRNA transcripts related to the regulation of transcription, RNA processing, and cellular stress response (71). Expression of ALS-linked mutations in FUS can shift the global transcriptome (18, 72). Specifically, a previous study reported that degradation of certain mRNA transcripts is increased following expression of the ALS-linked mutant FUS P525L (67). We also observed a positive interaction between the FUS variants and UPF1 and PABP1, both major mediators of the mRNA decay pathway, nonsense-mediated decay (NMD) (73, 74). For that reason, we asked whether expression of FUS PM altered the steady-state levels of specific mRNA transcripts regulated by NMD. We designed a quantitative PCR (qPCR) protocol to measure the total levels of the stress-related mRNA targets ATF3, ATF4, and TBL2 (Table S5). Total mRNA levels for UPF1 or FUS were unchanged between conditions (Fig. 5*A*). We report a significant increase in ATF3 mRNA levels in HEK293T cells expressing PM compared with EV, WT, and Δ15 (*p* = 0.0034, *p* = 0.0357, and *p* = 0.0008, respectively) (Fig. 5*B*). Furthermore, we observed a trend for an increase in ATF4 mRNA in HEK293T cells expressing Δ15 but not PM (EV *versus* Δ15, *p* = 0.3721; WT *versus* Δ15, *p* = 0.3994). We saw no difference in TBL2 mRNA levels following expression of FUS variants, suggesting not all stress-associated mRNAs are affected by FUS phosphorylation. Taken together, these data suggest that the steady-state levels of certain transcripts are increased by FUS PM expression.

Next, we set out to examine whether expression of FUS PM affected mRNA translation as the next highest enriched functional pathway was “translational assembly.” mRNA degradation is thought to be tightly coupled to translation because (1) translation requires multiple NMD factors, (2) phosphorylated UPF1 suppresses translational initiation, and (3) reinitiation of translation downstream of the premature termination codon can prevent NMD (73). Expression of FUS P525L was previously reported to decrease global protein translation (67). We utilized the SUrface SEnsing of Translation (SUnSET) assay to compare the amount of global protein synthesis between the FUS variants (75) (Fig. 5*C*). We saw a significant increase in the amount of protein synthesis in HEK293T cells expressing FUS PM compared with FUS WT (*p* = 0.0074) (Fig. 5*D*). Furthermore, we saw a trend toward a decrease in protein synthesis between PM and Δ15 (*p* = 0.0826) (Fig. 5*D*). Thus, protein translation is unchanged by Δ15 expression and enhanced by FUS PM expression compared with FUS WT.

Finally, we examined if FUS variants altered autophagosome formation because of the relationship of autophagy to “clathrin-mediated endocytosis” and the known role of lysosome/autophagy dysfunction in FTD and ALS pathogenesis (76). Clathrin-coated vesicles form the precursor phagophores, and blocking clathrin-dependent endocytosis leads to a decrease in autophagosome formation (77). Autophagosomes are double membrane vesicles that are integral to macroautophagy as they sequester cellular components and eventually fuse with acidic lysosomes to form autolysomes and degrade engulfed material (78, 79). We utilized an autophagic assay where we treated cells with bafilomycin (Baf), an inhibitor of the lysosomal V-ATPase, to block the fusion of autophagosomes leading to a build-up of autophagosomes (80). There was no difference in the levels of the autophagosome markers, LC3II and SQSTM1/p62, following expression of FUS variants, before or after Baf treatment (Fig. 5, *E*–*F*). Overall, these data suggest that while FUS PM expression affects early mRNA translation and regulation, it does not affect the total amount of autophagosomes or autophagosome flux.

## Discussion

Past studies have shown that FUS pathology leads to major changes in multiple functional pathways including (but not limited to) transcription patterns, splicing, DNA damage repair, translation, mRNA catabolism, and stress granule homeostasis (18, 24, 81, 82, 83, 84, 85). Proteomic analysis is a powerful tool that has revealed how pathogenic ALS-linked mutations (*e.g.*, FUS P525L and R495X) may lead to changes in these functional pathways (20, 54, 86). Past proteomic studies have utilized both targeted IPs and whole-cell analysis to map the proteome of cells expressing various toxic ALS-linked FUS mutations (*i.e.*, P525L, R495X) (54, 67, 86). Interactome level changes have been mapped for WT and ALS-linked R521G FUS (20). Furthermore, a recent study looked specifically at the interactome level changes in pathologically relevant droplets purified from WT and P525L FUS–expressing cell lysates (64).

While these past studies provide some insights into the role of *FUS* mutations on protein–protein interactions, pathogenic *FUS* mutations only account for ∼4% of ALS cases and a handful of FTD cases (32, 33, 87, 88). Thus, these previous studies do not address how nongenetic causes of FUS pathology, such as PTMs, may shift FUS function. Previous studies demonstrate that cytoplasmic accumulation of FUS can be triggered by other nongenetic mechanisms including loss of TNPO1–FUS interaction, cellular stressors, and/or altered PTMs (21, 34, 35, 36, 37, 38, 39, 48). Although the methylation state of FUS is altered in ALS/FTD-FUS postmortem tissue, the genetic causes, or cellular stress, have not been identified to explain this phenomenon (36, 89). In contrast to methylation, our laboratory has shown that a biologically relevant stressor, DSBs, triggers the DNA-PK to phosphorylate FUS at 12 key serine/threonine residues in the N-terminal SYGQ-low-complexity domain (44, 45, 46, 48, 57). Phosphorylated FUS then accumulates in the cytoplasm of the cell (46, 48). While previous studies have examined how DNA-PK–mediated N-terminal phosphorylation of FUS may shift the structure of the N terminus of FUS toward a more disordered state *in vitro*, none have determined whether phosphorylation at these residues alters the function of FUS in cells (44, 45). In this study, we investigated whether mimicking N-terminal phosphorylation at these 12 key residues alters protein interactome and function of FUS. We utilized the APEX2 system in combination with label-free proteomic analysis to investigate the role of N-terminal phosphorylation in the SYGQ-rich low-complexity domain on FUS function. Overall, this study is the first to map changes in the FUS protein interactome associated with a PTM.

The first question we aimed to address was whether the proteins enriched for APEX2-FUS PM overlap more with homeostatic APEX2-FUS WT or pathogenic APEX2-FUS P525L. From the 3349 proteins we identified in our study, 96.4% were shared between all three FUS variants (Fig. 2, *A*–*C*). This suggests that the pathogenic FUS P525L and the DSB-associated FUS PM variants may still interact (either directly or indirectly) with the majority of FUS WT targets (Fig. 2*A*). This is surprising as pathogenic variants of another ALS/FTD-linked protein, TDP-43, have been shown to interact with a large proportion of novel binding partners compared with WT TDP-43 (90, 91). One explanation may be that we used APEX2-mediated proximity labeling and captured a much larger set of interacting proteins. Nevertheless, the functional changes seen with FUS P525L and the DSB-associated FUS PM variants may not be due to the development of novel protein interactions but instead could be related to changes in the strength of interaction partners. For instance, methylation of key C-terminal residues in the RGG3 domain greatly shifts the strength of the interaction between FUS and its major nuclear import protein, TNPO1 (36, 92). In line with this, our data support the idea that FUS pathology is not because of a general loss of FUS interaction with target proteins since pathogenic FUS P525L interacted with most FUS WT target proteins (27, 93). These findings suggest that FUS pathogenesis may be due to changes in the strength of FUS interactions with other proteins.

To examine whether the strength of interactions between the FUS variants and protein hits differed, we focused on the top 10% most enriched protein hits for each variant and looked at the overlap of each group (Fig. 2*B*). Each sample clearly separated into three distinct groups (Fig. S2, *B* and *C*). This distribution suggests that while most of the protein interaction network is shared between the three groups, the datasets from APEX2-FUS WT and APEX2-FUS PM share more in common with each other than FUS P525L. If the protein-binding partners of FUS PM mirror FUS WT more than FUS P525L, does this indicate that expression of FUS PM does not alter the FUS interactome? To answer this question, we utilized differential expression analysis to directly examine the relative differences in abundance between the three groups. We saw that the comparison of APEX2-FUS WT and APEX2-FUS P525L exhibited the highest number of differentially expressed proteins followed by the comparison of APEX2-FUS PM and APEX2-FUS P525L (Fig. 2, *E*–*G*). From these comparisons, we generated lists of differentially enriched proteins to use in our downstream analysis.

We took advantage of the list of differentially enriched proteins between our groups to understand whether FUS function was affected by FUS PM expression. Past studies have demonstrated that expression of FUS P525L leads to functional changes in ontological pathways, including altered translation, altered splicing, and dysregulated chromatin (67, 83, 86, 94, 95). We show that APEX2-FUS P525L proximity biotinylated proteins were enriched in the cytoplasm, suggesting cytoplasmic functional pathways may be altered by FUS P525L expression (Fig. 1*H*) (29). In line with this, our APEX2-FUS P525L dataset was enriched for both cytoplasmic functional terms (“translation”) and structural terms (“actin filament–based process”), whereas depleted for nuclear terms related to mRNA (“spliceosome” and “regulation of mRNA processing”) and DNA processes (“covalent chromatin modification” and “DNA repair”). Accordingly, our FUS WT *versus* FUS P525L dataset agrees with previous functional studies demonstrating that the P525L mutation disrupts FUS localization, partially through impeding TNPO1-mediated nucleocytoplasmic shuttling (92, 96, 97).

The identified subgroup of enriched ontology terms for FUS PM over FUS WT was “mRNA catabolic process,” “translational assembly,” “stress granule assembly,” and “clathrin-mediated endocytosis.” These terms covered cytoplasmic functions consistent with the observation that FUS PM accumulates in the cytoplasm more than FUS WT (Fig. 1*D*). Even so, the role of N-terminal phosphorylation in FUS pathology is a debated topic. Other studies report N-terminal phosphorylation reduces the propensity of FUS to aggregate *in vitro*, thereby supporting a model where phosphorylation may be protective against cytoplasmic FUS–mediated toxicity (44, 57). In contrast, we provide evidence that N-terminal phosphorylation instead promotes the formation of FUS aggregates in cells (Fig. S4). Aggregation of FUS, independent of a pathogenic genetic mutation, may itself be sufficient to induce neurodegeneration (13). For this reason, aggregates of N-terminally phosphorylated FUS may be able to induce cellular toxicity. Future studies will need to investigate the role these FUS aggregates have in cellular heath.

Next, we utilized ProHits-viz to directly compare the abundance of the binding hits identified for four ontology terms between each FUS variants (Fig. 3). From this, we were able to visualize a multitude of proteins that overlap between ontology categories. We used these data along with the STRING interaction database to identify a subset of proteins from each ontology term that were either (1) previously identified binding partners for FUS WT (G3BP1, UPF1, PABP1, and eIF2α) or (2) a novel binding partner (MOV10) (20, 40, 67, 68, 70). As anticipated by the APEX2 datasets, we were able to confirm the interaction between all three FUS variants and the aforementioned targets utilizing two different methods: IP and IF (Fig. 4).

First, we confirmed that the GFP-tagged FUS PM and FUS P525L localized to the cytoplasm (Fig. 4). Furthermore, FUS PM and FUS P525L colocalized in the cytoplasm with the target proteins (G3BP1, UPF1, MOV10, and eIF2α (Fig. 4*C*). Even though FUS WT did not form distinct puncta or aggregates with these target proteins, it should be noted that FUS is a nucleocytoplasmic protein that shuttles between these two cellular compartments (25, 98). Therefore, while FUS WT accumulation in nuclear compartment is easily visualized through immunofluorescent staining, a significant portion of the protein is cytoplasmic (Fig. 1*C*). In support of this idea, we confirmed that all three FUS variants co-IP’d with previously identified binding partners (EWS, TAF-15, G3BP1, UPF1, PABP1, and eIF2α). We also confirmed a novel interaction between all three FUS variants and MOV10. Intriguingly, MOV10 has been previously linked to ALS–FTLD pathology. MOV10 is a member of the SF-1 RNA helicase family related to UPF1 and a component of the RNA-induced silencing complex (99). Exogenous expression of MOV10 was shown to ameliorate cell death in a TDP-43 model of ALS pathology (100). Our validation that these targets interact with FUS warrants future efforts to explore their role in FUS dysfunction and FTD–ALS pathogenesis.

Next, we set out to determine the extent that FUS PM expression affected functional pathways suggested by proximity labeling. Alterations in mRNA catabolic processing have been strongly linked to both ALS and FTD. One such process is NMD. NMD is a major cellular mechanism responsible for mRNA quality control by surveilling mRNA for premature termination codons (73, 101). UPF1 and PABP1, two proteins differentially enriched in FUS PM over FUS WT, act as opposing forces mediating the degradation/stabilization of NMD-sensitive mRNAs (102). A recent report found that NMD was inhibited in a C9orf72 model of FTD pathology, indicating that NMD dysfunction could be a common finding across the ALS–FTD spectrum (103). Overexpression of UPF1 in a model of FTD ameliorated toxicity in a model of ALS, suggesting enhancing NMD may be beneficial (104). In contrast, another report found that an ALS-linked FUS mutant enhanced NMD decay of targeted transcripts (67). What might explain these discrepancies? One possibility is that previous studies utilized model systems derived from different species. Studies that found diminished NMD were performed in human-derived models or using an *in vivo* mouse model of FUS pathology, whereas the study that shows enhanced NMD was done in an immortalized mouse cell line (67, 103, 105). Recently, we reported that mouse cells do not recapitulate DSB-mediated N-terminal phosphorylation of FUS (48), raising the possibility that FUS-mediated regulation of NMD is also not accurately recapitulated in mouse cells. To avoid these species-specific differences, we measured the steady-state levels of known targets of NMD using a qPCR assay in HEK293T cells. We found that mRNA transcript levels of ATF3, but not ATF4, are significantly increased following expression of FUS PM and truncated FUSΔ15 (Fig. 5*B*). These data suggest that expression of FUS PM may shift the steady-state levels of certain mRNA transcripts. Future studies will need to explore if NMD processes are responsible for this shift.

What might cause the divergence in steady-state ATF3 but not ATF4 transcript levels? Various cellular stressors such as the production of reactive oxygen species or endoplasmic reticulum stress leads to upregulation of ATF3 and ATF4 (106, 107). ATF3 is a stress-induced transcriptional activator associated with binding genomic sites related to cellular stress (108). In parallel, expression of ATF4 leads to ATP depletion, oxidative stress, and cell death (91). Upregulation of ATF4 occurs first, before directly inducing the expression of ATF3 and other downstream transcriptional regulators (106, 109). Given that we only assayed one time point, it is possible that while the 48-h time point captures the change in total transcript levels for ATF3, it may be too late to detect appreciable changes in ATF4 transcript levels. Furthermore, we did not detect altered transcript levels for another target of NMD, TBL2, or either (Fig. 5*B*) (106, 110). Thus, FUS PM expression may affect specific mRNA transcripts. Consistent with this idea, previous studies have shown that not all perturbations to the mRNA decay pathways equally affect transcript expression. For instance, depletion of NMD factor UPF2 enhanced ATF3 but not TBL2 mRNA transcript levels (110). Future studies should investigate the role of FUS phosphorylation on stress response pathways and the specificity of mRNA catabolic suppression on other transcripts.

FUS function is closely linked to regulation of mRNA translation (67, 81, 86, 111, 112). In line with this, we saw that expression of FUS PM enhanced protein translation compared with FUS WT (Fig. 5*D*). Interestingly, while we saw a trend, we did not find a significant change in protein synthesis following FUSΔ15 expression (Fig. 5*D*). Cytoplasmically localized ribonucleoprotein complex granules containing FUS, WT, or an ALS mutant have been reported to participate in active protein translation (112). Accordingly, FUS PM and FUS P525L in the cytoplasm may enhance protein translation through a similar mechanism. It should be noted that while the SUnSET assay is thought to reliably measure protein translation, it has limitations. First, the SUnSET assay measures relative rates of synthesis and is unable to capture the absolute changes, and second, differences in the amount of free puromycin between samples may alter puromycin uptake (75). Therefore, future studies should compare multiple methods of quantifying protein synthesis.

Finally, we examined how expression of FUS PM may impact autophagy through autophagosome formation. Lysosome-mediated autophagy is a multistage process involving multiple cellular components. In this process, autophagosomes are an integral part of the autophagy cascade where they begin as phagophores that expand into autophagosomes and fuse with endosomes and lysosomes to allow degradation of the compartment contents (77). Dysfunctional autophagosome formation and other aspects of the autophagy–lysosome pathway has been widely reported in ALS and FTD (76). In this study, we idented proteins involved in autophagosome formation such as CLTA in our APEX2 dataset. However, we did not detect any difference in the levels of two markers of autophagosomes following FUS WT and FUS PM expression, suggesting autophagosome formation is not affected in this assay (Fig. 5, *F* and *G*) (113). Nonetheless, it remains possible that phosphorylation of FUS, or expression of pathogenic *FUS* mutations, affects other aspects of autophagy and related pathways (*e.g.*, autophagic flux, lysosome health, fusion, endocytosis) (76, 114). Future studies should examine whether other parts of the clathrin-mediated endocytic pathway are affected by expression of FUS PM.

In conclusion, we report the first study examining whether a PTM, N-terminal phosphorylation, affects the FUS proteome. The use of APEX2 allowed us to generate a detailed map of the FUS interactome that included TNPO1 and TNPO2, which are known to import FUS into the nucleus. Importantly, we also identified novel nuclear import and export proteins in the FUS interactome, suggesting that the shuttling of FUS between the cytoplasm and nucleus is more complicated than previously appreciated, as supported by a recent publication (115). Furthermore, we identified a robust dataset of novel protein partners for FUS WT, FUS P525L, and a mimetic of N-terminal phosphorylation of FUS. Our data suggest that expression of phosphorylated FUS may impact cellular function by enhancing translation and suppressing mRNA degradation. These findings also shed light on fruitful avenues for future investigation. Future studies should examine how PTMs of FUS regulate protein function within the cell and how nongenetic factors influence processes underlying disease. The discovery that phosphorylated FUS may play a unique role in the mRNA homeostasis provides valuable insights into what functions may be dysregulated in the pathological cascades of ALS and FTD.

## Experimental procedures

### Plasmid generation

APEX2-FUS plasmids, maps, and sequences generated in this study are deposited in Addgene. The DNA sequences for the APEX2-FUS variants were designed *in silico* and then codon optimized and custom synthesized by GenScript. The amino acid sequence for the engineered APEX2 was taken from Addgene plasmid #212574. The WT FUS sequence was taken from National Center for Biotechnology Information reference sequence RNA-binding protein FUS isoform 1 (*Homo sapiens*) (NP_004951.1). A Twin-Strep tag was added to the N terminus of the APEX2 sequence. A linker region (GGGS)^3^ with an FLAG tag (DYKDDDDK) was included at the end of APEX2 followed by the FUS sequence. Synthetic APEX2-FUS gene constructs were designed to add a 5′ BamHI restriction digestion site (GGATCC) followed by a Kozak sequence (GCCACC) before the ATG start codon of APEX2, a 3′ stop codon (TAG), and an ending with a XhoI restriction digestion site (CTCGAG). Following synthesis, the APEX2-FUS WT fusion protein was inserted into the pcDNA3.1/Hygro(+) vector using a BamHI/XhoI cloning strategy. The APEX2-FUS P525L and APEX2-FUS PM constructs were generated from the donor APEX2-FUS WT construct by express mutagenesis (GenScript).

The GFP-tagged FUS variants were designed by adding enhanced GFP (EGFP) to the N terminus of the previously described FUS variants in the study by Deng *et al*. (46). In brief, the FUS variants (WT, Ala sub, PM, and Δ15) were synthesized and ligated into pcDNA3.1(+) Hygro by GeneArt (Thermo Fisher Scientific). The FUSΔ15 variant was engineered to introduce a stop codon at serine 513 leading to a truncated protein lacking the amino acids encoded by exon 15 (termed FUS S513X or FUSΔ15), which completely lacks the C-terminal nuclear localization signal. These constructs were then digested at NheI/HindIII sites upstream of the FUS sequence. EGFP was PCR amplified to introduce an NheI restriction site at the 5′ end and a HindIII site at the 3′ end. The EGFP was then digested and ligated into each construct. The primers used to generate EGFP were: GFP.Nhe.Sense

(CACTATAGGGAGACCCAAGCTGGCTAGCgccaccATGGTGAGCAAGGGCGAGGAGCTG) and GFP.Hind.Antisense:

(GGGACCAGGCGCTCATGGTGGCAAGCTTCTTGTACAGCTCGTCCATGCCGAG).

The GFP-tagged FUS P525L variant was created by site-directed mutagenesis on the GFP-tagged FUS WT construct using the QuikChange II XL Site-Directed Mutagenesis Kit (Agilent; catalog no.: 200521). The primers used to generate the construct were:

P525L_Sense (gacagaagagagaggctctactgactcgagtct)

P525L_Antisense (agactcgagtcagtagagcctctctcttctgtc)

All constructs were verified using DNA sequencing, restriction digests, and/or PCR amplification. The full DNA sequence for each synthesized sequence can be found in Table S4.

### Cell culture

HEK293T (American Type Culture Collection) cells were cultured in Dulbecco's modified Eagle's medium supplemented with 10% fetal bovine serum (Atlanta Biological) and 1% penicillin/streptomycin (Gibco). Cells were maintained at 37 °C with 5% CO_2_.

### Cell transfection and APEX2-mediated biotinylation

HEK293T cells were seeded onto a poly-l-lysine–coated 10-cm cell culture grade dish and cultured for 2 days prior to transfection. Cells were transfected at ∼60% confluency with 2.5 μg of the appropriate DNA construct using the TransIT-LT1 Transfection Reagent (Mirus; catalog no.: MIR2300) and cultured for an additional 2 days. At ∼48 h post-transfection, 500 μM biotinyl tyramide (biotin phenol) (Tocris; catalog no.: 6241) supplemented in Dulbecco's modified Eagle's medium with 10% fetal bovine serum/1% penicillin/streptomycin was added to all experimental plates except for the nontransfected control plates. Labeling was initiated after 30 min by adding H_2_O_2_ (1 mM final concentration) for 1 min. The labeling reaction was quenched by aspirating media from the plate and immediately rinsing three times with the quenching solution: 5 mM trolox ((+/−)-6-hydroxy-2,5,7,8-tetramethylchromane-2-carboxylic acid; Sigma [catalog no.: 238813]), 10 mM sodium l-ascorbate (Sigma; catalog no.: A4034), and 10 mM sodium azide in PBS supplemented with 1× PMSF, a serine protease inhibitor. Cells were then incubated on ice in fresh quenching solution four times for 5 min each. Following the last wash, the quenching solution was aspirated off, and 600 μl cold lysis buffer (50 mM Tris, 150 mM NaCl, 0.4% SDS, 0.5% sodium deoxycholate, 1% Triton X-100, 10 mM sodium azide, 10 mM sodium ascorbate, and 5 mM Trolox) supplemented with 1× Halt protease/phosphatase inhibitor (Thermo Fisher Scientific; catalog no.: 78446) was added to each plate. Samples were collected with cell scrapers into Protein lo-bind tubes (Eppendorf) and sonicated 2× on ice (25 amplitude: 10 s total on ice, 2 s on/2 s off). Samples were cleared by centrifugation at 16,500*g* for 10 min at 4 °C, and the supernatant was collected into fresh protein lo-bind tubes. About 540 μl of prechilled 50 mM Tris (pH = 7.4) was added to wash each pellet, and samples were spun at 16,500*g* for 10 min at 4 °C. Supernatant was collected and combined to previous samples, and samples were stored at −80 °C. Protein concentration was assayed using RC DC protein assay (Bio-Rad; catalog no.: 5000121).

### Streptavidin-based purification of biotinylated targets

For affinity purification, 240 μl of NanoLINK Streptavidin Magnetic Beads (TriLink Biotechnologies; catalog no.: M-1002) were washed 3× in 1× Tris-buffered saline (TBS) containing 0.1% Tween-20 (TBST). About 1.8 mg of total protein was then added onto washed beads and allowed to incubate overnight at 4 °C with mixing. Beads were then collected against a magnetic stand, and the supernatant was set aside for future analysis (termed flow-through). Beads were then washed in wash buffer 1 (50 mM Tris, 150 mM NaCl, 0.4% SDS, 0.5% sodium deoxycholate, and 1% Triton X-100) and gently mixed with rotation for 5 min at room temperature (RT). Supernatant was discarded. Beads were then washed in wash buffer 2 (2% SDS in 50 mM Tris–HCl, pH 7.4) and gently mixed with rotation for 5 min at RT. Supernatant was discarded. Beads were then washed 2× in wash buffer 1 with rotation for 5 min at RT. About 10% of bead slurry from each sample was set aside for future analysis (termed elution). Remaining beads were then washed 4× in 1× PBS and stored at −20 °C.

### On-bead digestion and label-free MS

Urea (8 M) was added to the beads, and the mixture was then treated with 1 mM DTT at RT for 30 min, followed by 5 mM iodoacetimide at RT for 30 min in the dark. Proteins were digested with 0.5 μg of lysyl endopeptidase (Wako) at RT for 4 h and further digested overnight with 1 μg trypsin (Promega) at RT. Resulting peptides were desalted with HLB column (Waters) and dried under vacuum.

### MS

The data acquisition by LC–MS/MS was adapted from a published procedure (116). Derived peptides were resuspended in the loading buffer (0.1% TFA). Peptide mixtures were separated on a self-packed C18 (1.9 μm, Dr Maisch HPLC GmbH) fused silica column (50 cm × 75 μm internal diameter; New Objective) attached to an EASY-nLC 1200 system and were monitored on a Q-Exactive Plus Hybrid Quadrupole-Orbitrap Mass Spectrometer (Thermo Fisher Scientific). Elution was performed over a 106 min gradient at a rate of 300 nl/min (buffer A: 0.1% formic acid in water; buffer B: 0.1% formic acid in acetonitrile): The gradient started with 1% buffer B and went to 7% in 1 min, then increased from 7% to 40% in 105 min, then to 99% within 5 min, and finally staying at 99% for 9 min. The mass spectrometer cycle was programmed to collect one full MS scan followed by 20 data-dependent MS/MS scans. The MS scans (*m/z* range of 350–1500, 3 × 10^6^ automatic gain control target, 100 ms maximum ion time) were collected at a resolution of 70,000 at *m/z* 200 in profile mode. The higher energy collision dissociation MS/MS spectra (*m/z* 2 isolation width, 28% collision energy, 1 × 10^5^ automatic gain control target, and 50 ms maximum ion time) were acquired at a resolution of 17,500 at *m/z* 200. Dynamic exclusion was set to exclude previously sequenced precursor ions for 30 s within a 10 ppm window. Precursor ions with +1, and +8, or higher charge states were excluded from sequencing.

### Proteomic data processing

#### Raw data processing

Raw files were processed by MaxQuant with default parameters for LFQ (117). MaxQuant employs the proprietary MaxLFQ algorithm for LFQ. Quantification was performed using razor and unique peptides, including those modified by acetylation (protein N-terminal), oxidation (Met), and deamidation (NQ). Spectra were searched against the Human UniProt database (90,300 target sequences). The resulting data with intensity scores were run through the SAINT software (version 2.5) to identify and remove proteins that were unlikely to be true bait–prey interactions (61). This was performed by comparing protein intensity values in the negative control condition to the corresponding intensity values in the samples. Proteins with less than 95% probability to be significantly different from the negative control in all samples were removed. The mean intensity values of control were subtracted from each sample intensity value for the remaining proteins.

#### Statistical analysis

The resulting protein group information was read in R and analyzed using Proteus to determine differentially expressed proteins between groups (118). LFQ intensities of each sample were log_2_ transformed and compared using a linear model with standard errors smoothed by empirical Bayes estimation, taken from the R package limma, to determine differentially enriched proteins. Nominal *p* values were transformed using the Benjamini–Hochberg correction to account for multiple hypothesis testing (119). Proteins were considered significantly differentially enriched if they had *q* values less than 0.01 and an absolute value of log_2_ FC greater than 1, or twice as enriched linearly.

Data quality was assessed through distance matrices and principal component analysis. Volcano plots were custom generated but drew heavily from thematic elements from the R package Enhanced Volcano (https://bioconductor.org/packages/devel/bioc/vignettes/EnhancedVolcano/inst/doc/EnhancedVolcano.html#references, accessed July 1, 2020). Pathway overrepresentation analysis was performed using MetaScape with default settings (62). Pathway overrepresentation *p* values were adjusted using the Benjamini–Hochberg correction, and significant pathways were determined from those with *q* values less than 0.01. Biologically interesting pathways were selected manually, and the gene sets that constituted those pathways were submitted to ProHitz-viz dot plot generator to view protein-level enrichment differences for the selected pathways (63). In the ProHitz dot plots, the rows were sorted by hierarchical clustering using Canberra distance and Ward’s minimum variance method for clustering. The columns were sorted manually. Venn diagrams for overlapping proteins across the conditions were generated using the R packages ggvenn or ggVennDiagram (https://cran.r-project.org/web/packages/ggvenn/index.html, accessed July 1, 2020, 120). The heatmap was generated using the R package pheatmap (https://cran.r-project.org/web/packages/pheatmap/index.html, accessed February 2, 2021). The GO summary table (Table 1) was generated using R package gt (https://cran.r-project.org/web/packages/pheatmap/index.html, accessed January 29, 2021).

### IF

About 24 h post-transfection, cells were washed three times at RT with and fixed in 4% paraformaldehyde for 15 min. After washing, cells were permeabilized in 0.5% Triton X-100 for 10 min. Cells were then washed three times in either 1× Dulbecco’s PBS (DPBS) or 1× TBS and blocked in 3% bovine serum albumin for 1 h at RT. After blocking, cells were incubated overnight at 4 °C in primary antibody diluted in blocking buffer. The next day cells were washed three times with DPBS or TBS and incubated in secondary antibody diluted 1:500 or 1:750 in blocking buffer (Cy5 Donkey anti-rabbit, catalog no.: 711-175-152; Cy5 Donkey antimouse, catalog no.: 715-175-151; and 488 Goat antimouse, catalog no.: A-11029). Following incubation, cells were washed three times in DPBS or TBS and mounted onto glass slides using Prolong Gold with 4′,6-diamidino-2-phenylindole (Thermo Fisher Scientific; catalog no.: P36935). The following primary antibodies were used: UPF1 (Cell Signaling Technologies; catalog no.: 12040S; 1:2000 dilution), MOV10 (Proteintech; catalog no.: 10370-1-AP; 1:1000 dilution), eIF2α (Cell Signaling Technologies; catalog no.: 9722S; 1:500 dilution), G3BP1 (Proteintech; catalog no.: 13057-2-AP; 1:2500 dilution), PABP1 (Cell Signaling Technologies; catalog no.: 4992S; 1:500 dilution), Twin-Strep tag (IBA Lifesciences; catalog no.: 2-1517-001; 1:1000 dilution), and Streptavidin 660 Conjugate (Thermo Fisher Scientific; catalog no.: S21377; 1:500 dilution). Images were collected on a Leica DMi8 THUNDER Inverted Fluorescence Microscope with a DFC7000 T camera (Leica).

### IP

About 24 h post-transfection, cells were washed two times on ice with DPBS. Cells were scraped in 1 ml of PBS and spun at 500*g* for 5 min at 4 °C. From this point on, cells were processed as previously described with slight modification (121). In brief, cells were lysed in 10 mM Tris–HCl, pH 7.4, 5 mM MgCl_2_, 10 mM NaCl with 1× cOmplete protease inhibitor cocktail (PIC) (Millipore Sigma; catalog no.: 11873580001) and spun at 1200*g* for 5 min at 4 °C. The postnuclear supernatant (PNS) was collected and labeled the cytoplasmic faction. The pellet was sonicated and subjected to successive rounds of lysis and centrifugation in HGN 165 buffer (10 mM Hepes–NaOH, pH 7.25, 10% glycerol [v:v], 165 mM NaCl), 1% Triton X-100, 2 mM MgCl_2_, 1 mM DTT, benzonase (250 U/μl), 4× cOmplete PIC, with increasing concentrations of Triton X-100 and sodium chloride. The resulting four nuclear lysates were then pooled and either combined with the PNS to generate a whole-cell lysate (Fig. 4*A*) or kept separate from the PNS (Fig. 4*B*) and immunoprecipitated with MagStrep Type3 beads (IBA Lifesciences; catalog no.: 2-4090-002) overnight with end/end rocking at 4 ˚C. Beads were washed 3× in HGN 165 buffer with 1× PIC cocktail. Bound material was eluted in 0.1 M Tris–HCl (pH 8.0), 0.15 M NaCl, 0.05 M biotin at RT for 15 min, followed by a second elution in nonreducing 2× Laemmli buffer at RT for 15 min, and 95 °C for 5 min. Elutions were pooled, and 1% of the original input was reduced and denatured for SDS-PAGE and Western blotting as described later.

### Cytoplasmic/nuclear fractionation

Cell lysis was performed as previously described with minor modifications (46, 48). In brief, cells were lysed on ice in cytoplasmic lysis buffer (50 mM Tris [pH = 8.0], 150 mM NaCl, and 0.5% Triton X-100) with 1% protein/phosphatase inhibitor (Thermo Fisher Scientific; catalog no.: 78442). The cell suspension was centrifuged at 14,000 rpm for 15 min at 4 °C. The supernatant was transferred to a new tube and labeled the cytoplasmic fraction. Remaining cell pellet was washed 2× in cytoplasmic lysis buffer, and supernatant was discarded. Pellet was then resuspended in radioimmunoprecipitation assay (RIPA) buffer (50 mM Tris [pH = 8.0], 150 mM NaCl, 0.1% SDS, 1% Triton X-100, and 0.5% sodium deoxycholate) with 1% protein/phosphatase inhibitor (Thermo Fisher Scientific; catalog no.: 78442), and the suspension was sonicated and then clarified by centrifugation at top speed. Supernatant was transferred to a new tube and labeled the nuclear fraction. Subcellular fractionation was confirmed *via* Western blot showing isolation of GAPDH and H3 to cytoplasm and nucleus, respectively.

### Western blot

Cell lysis and Western blotting was performed as previously described with minor modifications (48). In brief, cells were lysed on ice in either RIPA buffer (50 mM Tris [pH = 8.0], 150 mM NaCl, 0.1% SDS, 1% Triton X-100, and 0.5% sodium deoxycholate) or cytoplasmic lysis buffer (50 mM Tris [pH = 8.0], 150 mM NaCl, and 0.5% Triton X-100) with 1% protein/phosphatase inhibitor (Thermo Fisher Scientific; catalog no.: 78442). The RIPA lysate was sonicated and centrifuged for 15 min at 14,000 rpm at 4 °C. The cytoplasmic lysate was vortexed and centrifuged for 15 min at 14,000 rpm at 4 °C. The supernatant was saved as the detergent-soluble protein fraction. Protein concentrations were measured in the detergent-soluble protein fraction by bicinchoninic assay (Pierce). Next, cell lysates were analyzed for relative protein expression using SDS-PAGE followed by two-channel infrared quantitative Western blots as described previously (46). The samples were denatured in 1× Laemmli loading buffer with 5% Tris(2-carboxyethyl) phosphine at 70 °C for 15 min. Equal amounts of protein were loaded into a 4 to 20% PROTEAN TGX Precast Gels (Bio-Rad). After transferring to 0.2 μm nitrocellulose membranes, some blots were stained with Revert 700 (LI-COR; catalog no.: 926-11010) to measure total protein for normalization, and signal was captured at 700 nm on an Odyssey Fc Imaging System (LI-COR), and then destained following the manufacturer's protocol. Protein blots were then blocked in EveryBlot Blocking Buffer (Bio-Rad; catalog no.: 12010020) for 5 min at RT and incubated with primary antibodies (diluted in blocking buffer) overnight at 4 °C. Membranes were washed three times for 5 min in TBST and then incubated with the appropriate secondary antibody diluted in blocking buffer for 60 min at RT. Finally, membranes were washed three times with TBST for 5 min and visualized using the Odyssey Fc Imaging System (LI-COR). The following primary antibodies were used: StrepMAB-Immo (anti–Twin-Strep tag; IBA Lifesciences; catalog no.: 2-1517-001; 1:4000 dilution), FUS (Bethyl Laboratories; catalog no.: A300-302A; 1:2000 dilution), UPF1 (Cell Signaling Technologies; catalog no.: 12040S; 1:1000 dilution), MOV10 (Proteintech; catalog no.: 10370-1-AP; 1:800 dilution), eIF2α (Cell Signaling Technologies; catalog no.: 9722S; 1:500 dilution), G3BP1 (Proteintech; catalog no.: 13057-2-AP; 1:2000 dilution), PABP1 (Cell Signaling Technologies; catalog no.: 4992S; 1:1000 dilution), G3BP1 (Proteintech; catalog no.: 13057-2-AP; 1:2000 dilution), TAF-15 (Bethyl Laboratories; catalog no.: A300-308A); EWS (Epitomics; catalog no.: 3319-1; 1:1000 dilution), Anti-Puromycin (Sigma–Aldrich; catalog no.: MABE343; 1:5000 dilution), LC3A/B (Cell Signaling Technologies; catalog no.: 12741; 1:1000 dilution), SQSTM1/p62 (Cell Signaling Technologies; catalog no.: 5114; 1:1000 dilution), GAPDH (Cell Signaling Technologies; catalog no.: 2118; 1:10,000 dilution), and H3 (Millipore; catalog no.: 06-599; 1:5000 dilution).

### qPCR

About 48 h post-transfection, cells were harvested for RNA using TRIzol Reagent (Thermo Fisher Scientific; catalog no.: 15596026) following manufacturer guidelines. Equal amounts of RNA were used to create the complementary DNA library using the High-Capacity cDNA Reverse Transcription Kit with RNase Inhibitor (Thermo Fisher Scientific; catalog no.: 4374966). qPCR was performed on a CFX96 Touch Real-Time PCR Detection System (Bio-Rad) using the PowerUp SYBR Green Master Mix (Thermo Fisher Scientific; catalog no.: A25741). Results were quantified using the ΔΔCT method. Primers are listed in Table S5.

### SUnSet assay

Puromycin was obtained from Gibco suspended in 20 mM Hepes (pH 6.7). Drug was aliquoted and stored at −20 °C (Thermo Fisher Scientific; catalog no.: A1113803). About 48 h post-transfection, cells were treated with 1 μM puromycin diluted in cell culture media for 30 min at 37 °C/5% CO_2_. Control cells were treated with vehicle (20 mM Hepes [pH 6.7]) diluted in cell culture media for 30 min at 37 °C/5% CO_2_. Following treatment, cells were lysed in RIPA lysis buffer + 1% protein/phosphatase inhibitor and subjected to SDS-PAGE and Western blotting as described previously.

### Autophagosome assay

Baf A1 was obtained from Tocris (catalog no.: 1334) and resuspended in dimethyl sulfoxide and aliquoted and stored at −20 °C. About 48 h post-transfection, cell were treated with 0.1 μM Baf diluted in cell culture media for 4 h at 37 °C/5% CO_2_. Control cells were treated with vehicle (dimethyl sulfoxide) diluted in cell culture media for 4 h at 37 °C/5% CO_2_. Following treatment, cells were lysed in RIPA lysis buffer + 1% protein/phosphatase inhibitor and subjected to SDS-PAGE and Western blotting as described previously.

### Statistical analysis

Nonproteomic statistical analysis was performed using GraphPad Prism 8 (GraphPad Software, Inc). Effect of variant on FUS localization was determined using an ordinary one-way ANOVA with Tukey's post hoc test (Fig. 1, *C* and *D*). Effect of variant on UPF1 mRNA FC expression was determined using an ordinary one-way ANOVA with Tukey’s post hoc test (Fig. 5*A*). Effect of variant on mRNA FC for other targets was determined using a two-way ANOVA with Tukey’s post hoc test (Fig. 5*B*). Effect of variant on autophagosome markers was determined using a mixed model two-way ANOVA with Tukey’s post hoc test (Fig. 5, *F* and *G*). Significance was reached at *p* < 0.05. Significance is designated as *p* < 0.05 (∗), *p* ≤ 0.0021 (∗∗), *p* ≤ 0.0002 (∗∗∗), and *p* ≤ 0.0001 (∗∗∗∗). All quantified blots either normalized to total protein (Figs. 1*E* and 5, *D*–*G*), GAPDH (Fig. 1, *D* and *E*), or H3 (Fig. 1, *D* and *E*).

## Data availability

The APEX2 MS proteomic data from this publication have been deposited to the ProteomeXchange Consortium *via* the PRIDE partner repository (https://www.ebi.ac.uk/pride/archive/) and assigned the dataset identifier PXD026578 (122).

## Supporting information

This article contains supporting information (Figs. S1–S4 and Tables S1–S5).

## Conflict of interest

The authors declare that they have no conflicts of interest with the contents of this article.

## Supporting information

Supporting Table 1 APEX2FUS proteomics dataset

Supporting Table 2 Differential enrichment

Supporting Table 3 Other FUS proteomes

Supporting Table 4 Plasmid Sequences

Supporting Table 5 qPCR primers
