# Supplementary material for: Proximity-based labeling reveals DNA damage–induced phosphorylation of fused in sarcoma (FUS) causes distinct changes in the FUS protein interactome
Source: J Biol Chem. 2022 Jun 14;298(8):102135. doi: 10.1016/j.jbc.2022.102135 (PMC9372748; doi:10.1016/j.jbc.2022.102135)
Supplement: Supporting Figure 1 [file mmc6.pdf]

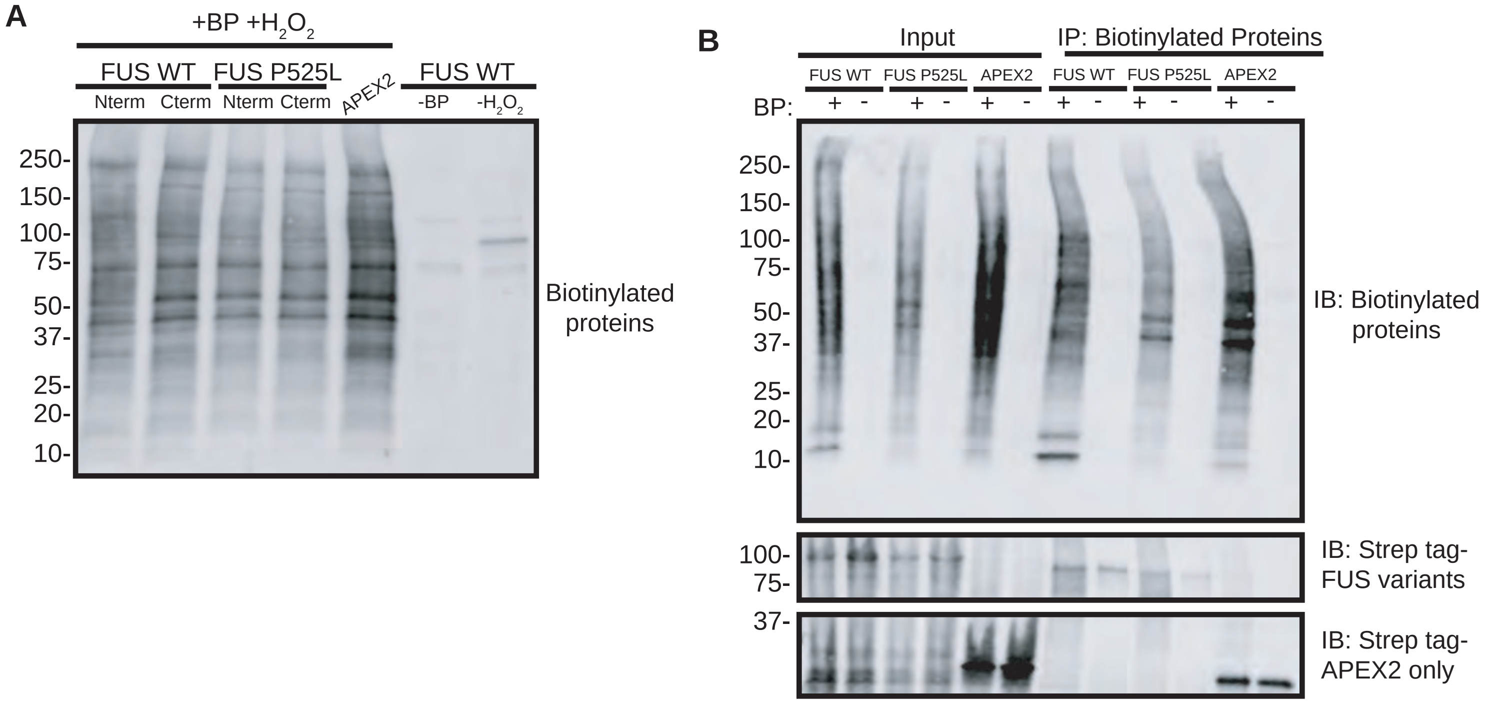
A
+BP +H2O2
FUS WT
FUS P525L
Nterm
Cterm
Nterm
Cterm
APEX2
FUS WT
-BP
-H2O2
250-
150-
100-
75-
50-
37-
25-
20-
10-
Biotinylated proteins
B
Input
IP: Biotinylated Proteins
FUS WT
FUS P525L
APEX2
FUS WT
FUS P525L
APEX2
BP: + - + - + - + - + - + -
250-
150-
100-
75-
50-
37-
25-
20-
10-
IB: Biotinylated proteins
100-
75-
IB: Strep tag-FUS variants
37-
IB: Strep tag-APEX2 only

Supplemental Figure 1. Cells expressing APEX2-FUS fusion constructs need biotin-phenol (BP) and H_2O_2 to induce biotinylation.

(A) Western blot of cell lysate from HEK293T cells expressing different APEX2-FUS fusion constructs. Constructs either had APEX2 fused to the N-terminus of the FUS variant (Nterm) or the C-terminus of the FUS variant (Cterm) or did not have FUS fused to APEX2 (APEX2). Following transfection, cells were 1) given biotin-phenol (BP) and H_2O_2 (+BP + H2O2), 2) given only H_2O_2 (-BP) or 3) given only BP (-H_2O_2). 24 hours post-transfection, biotinylation was induced, quenched and cells lysate was harvested and analyzed for biotinylated proteins (detected with streptavidin). (B) Immunoprecipitation of biotinylated proteins using streptavidin magnetic beads. Biotinylated proteins are only pulled down when cells were given biotin-phenol (BP). Input is 10% of sample loaded onto magnetic beads coated with streptavidin; Elute is 100% of sample eluted off beads. Samples are from cells expressing the following APEX2-FUS fusion proteins: wildtype FUS (FUS WT), P525L FUS (FUS P525L), and APEX2 without FUS fusion (APEX2). Input and elution were analyzed for biotinylated proteins (streptavidin) and Twin-Strep-tag® (StrepImmo antibody).
