# Supplementary material for: Proximity-based labeling reveals DNA damage–induced phosphorylation of fused in sarcoma (FUS) causes distinct changes in the FUS protein interactome
Source: J Biol Chem. 2022 Jun 14;298(8):102135. doi: 10.1016/j.jbc.2022.102135 (PMC9372748; doi:10.1016/j.jbc.2022.102135)
Supplement: Supporting Figure 2 [file mmc7.pdf]

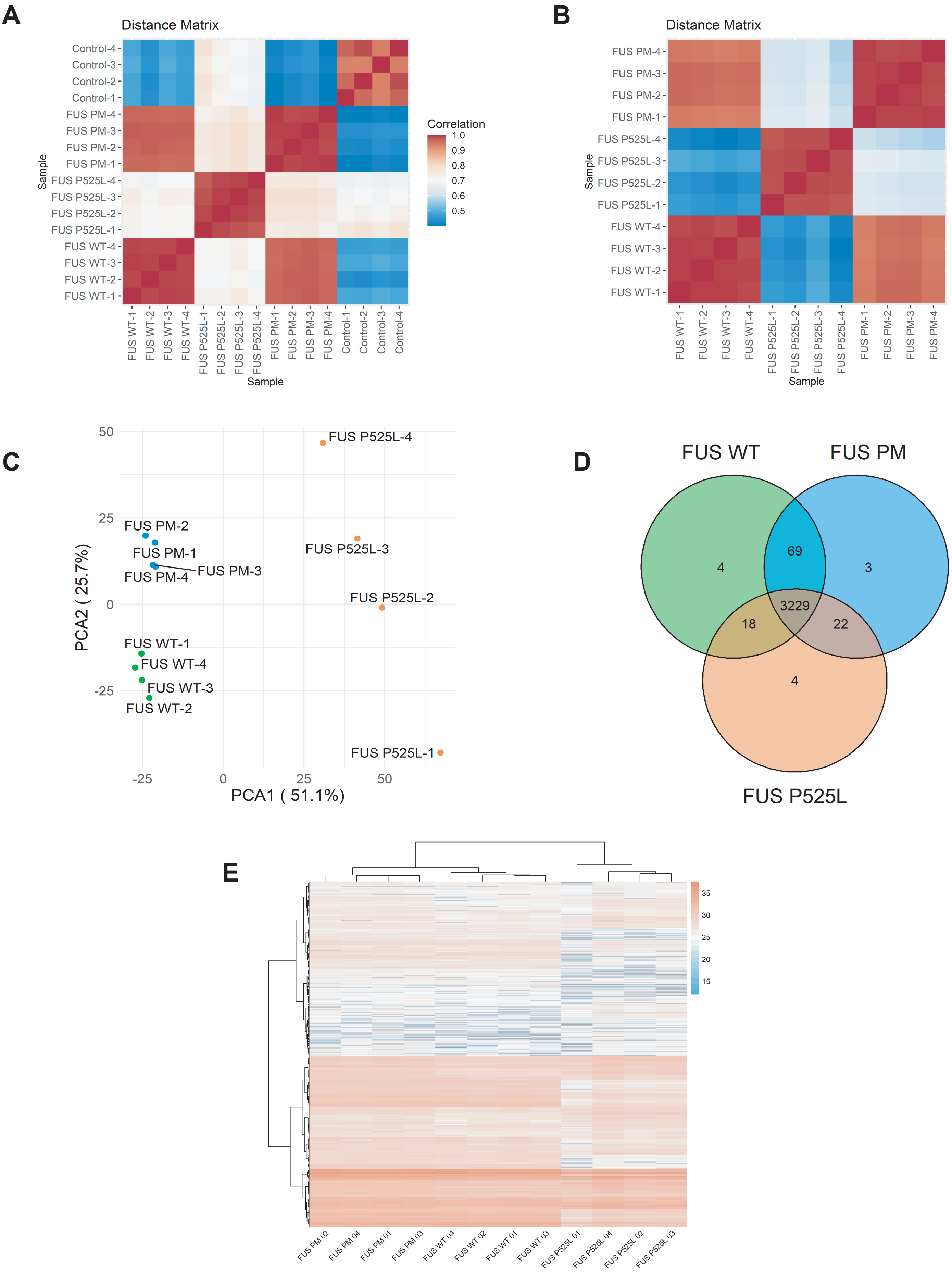
A
Distance Matrix
Control-4
Control-3
Control-2
Control-1
FUS PM-4
FUS PM-3
FUS PM-2
FUS PM-1
FUS P525L-4
FUS P525L-3
FUS P525L-2
FUS P525L-1
FUS WT-4
FUS WT-3
FUS WT-2
FUS WT-1
Sample
FUS WT-1
FUS WT-2
FUS WT-3
FUS WT-4
FUS P525L-1
FUS P525L-2
FUS P525L-3
FUS P525L-4
FUS PM-1
FUS PM-2
FUS PM-3
FUS PM-4
Control-1
Control-2
Control-3
Control-4
Sample
Correlation
1.0
0.9
0.8
0.7
0.6
0.5
B
Distance Matrix
FUS PM-4
FUS PM-3
FUS PM-2
FUS PM-1
FUS P525L-4
FUS P525L-3
FUS P525L-2
FUS P525L-1
FUS WT-4
FUS WT-3
FUS WT-2
FUS WT-1
Sample
FUS WT-1
FUS WT-2
FUS WT-3
FUS WT-4
FUS P525L-1
FUS P525L-2
FUS P525L-3
FUS P525L-4
FUS PM-1
FUS PM-2
FUS PM-3
FUS PM-4
Sample
C
50
25
0
-25
PCA2 (25.7%)
FUS P525L-4
FUS PM-2
FUS P525L-3
FUS PM-1
FUS PM-3
FUS PM-4
FUS P525L-2
FUS WT-1
FUS WT-4
FUS WT-3
FUS WT-2
FUS P525L-1
-25
0
25
50
PCA1 (51.1%)
D
FUS WT
FUS PM
4
69
3
3229
18
22
4
FUS P525L
E
35
30
25
20
15
FUS PM 02
FUS PM 04
FUS PM 01
FUS PM 03
FUS WT 04
FUS WT 02
FUS WT 01
FUS WT 03
FUS P525L 01
FUS P525L 04
FUS P525L 02
FUS P525L 03

Supplemental Figure 2. APEX2-FUS variant proteomes have unique signatures.

(A) Distance matrix of Control, FUS PM, FUS WT, FUS P525L samples. Pearson correlation between samples within and between groups with red indicating values closer to 1.0 and blue indicating values closer to 0.5. (B) Distance matrix of all samples, following normalization to control samples, showing the Pearson correlation between samples within and between groups with red indicating values closer to 1.0 and blue indicating values closer to 0.5. (C) Principal Component Analysis (PCA), excluding controls, showing reproducibility of data between biological replicates in FUS PM, FUS WT, FUS P525L samples. (D) Venn diagram of overlap and unique protein hits identified in APEX2-FUS WT, FUS PM, and FUS P525L proteomes. (E) Hierarchical clustering of samples based on the intensity profiles of all proteins identified. Missing values are colored gray.
