# Supplementary material for: Proximity-based labeling reveals DNA damage–induced phosphorylation of fused in sarcoma (FUS) causes distinct changes in the FUS protein interactome
Source: J Biol Chem. 2022 Jun 14;298(8):102135. doi: 10.1016/j.jbc.2022.102135 (PMC9372748; doi:10.1016/j.jbc.2022.102135)

Supplemental Figure 3. Graphical representation of protein enrichment for top significantly enriched gene ontology (GO), reactome, and pathways.

Dot plots were generated using Prohits-viz and are a graphical representation of the relative binding intensity of each protein against the three FUS variant groups.

Table of Contents:

A. Figure legend
B. GO:0030029 Actin filament-based process
C. GO:0016569 covalent chromatin modification
D. GO:0016482 cytosolic transport
E. GO:0006281 DNA repair
F. FET proteins + TARDBP
G. GO:0048193 Golgi Vesicle Transport
H. R-HSA-199991 Membrane trafficking
I. GO:0120031 plasma membrane bounded cell projection assembly
J. GO:0022613 Ribonucleoprotein complex biogenesis
K. CORUM:351 Splicesome
L. GO:0006412 Translation
M. GO:0050684 regulation of mRNA processing

3A. Figure legend

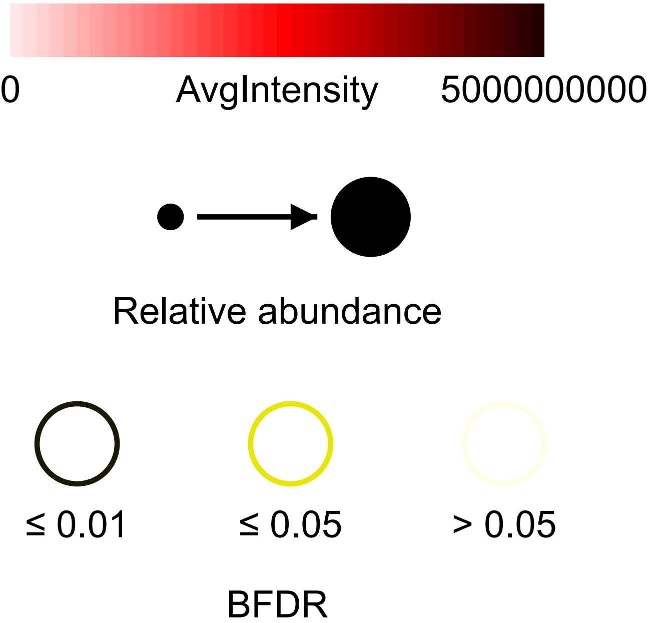

3B. GO:0030029 Actin filament-based process

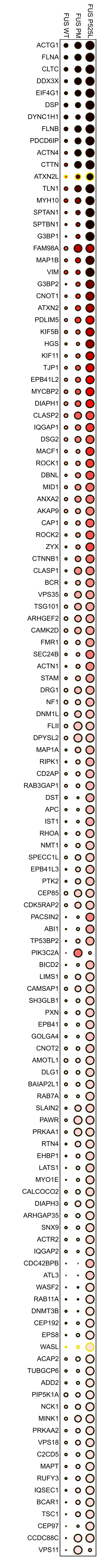

3C. GO:0016569 covalent chromatin modification

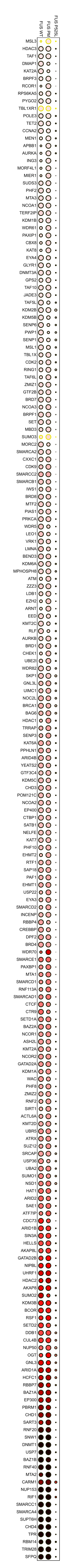

3D. GO:0016482 cytosolic transport

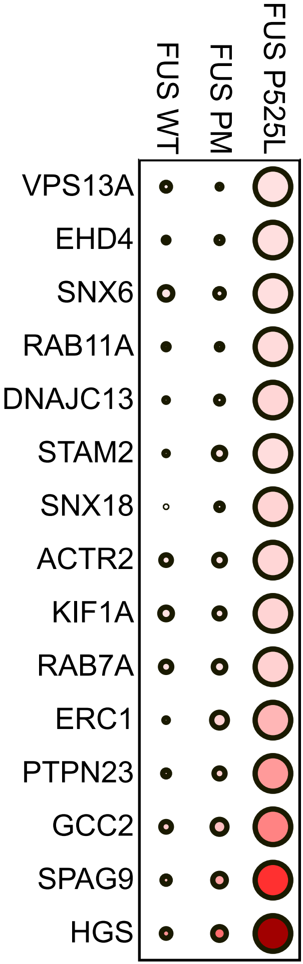

3D. GO:0006291 DNA repair

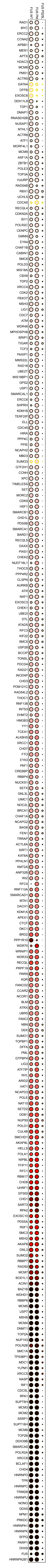

3F. FET proteins + TARDBP

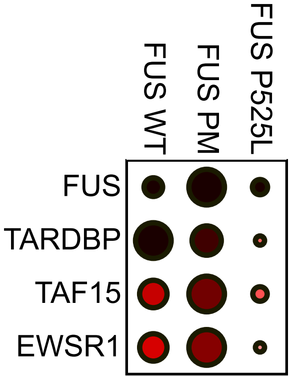

3G. GO:0048193 Golgi Vesicle Transport

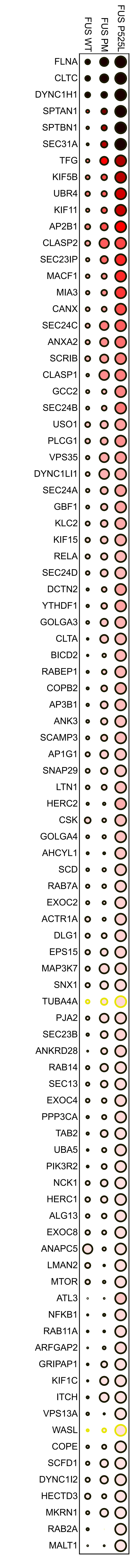

3H. R-HSA-199991 Membrane trafficking

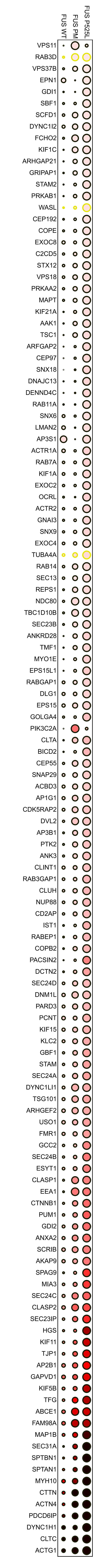

3I. GO:0120031 plasma membrane bounded cell projection assembly

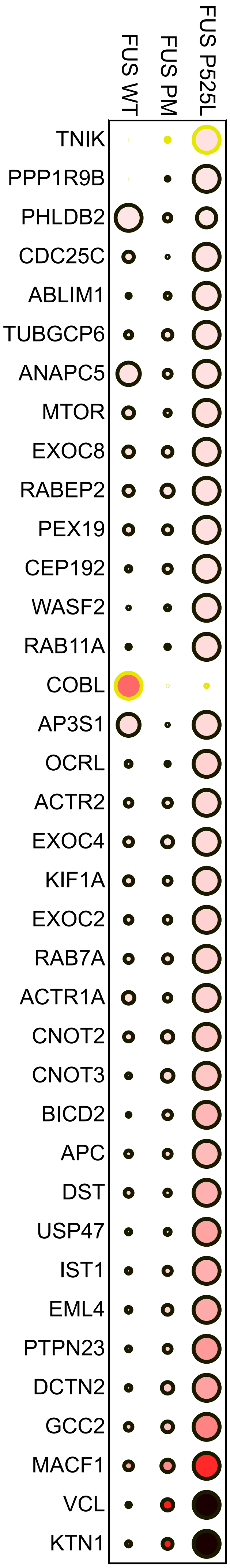

3J. GO:0022613
Ribonucleoprotein complex
biogenesis

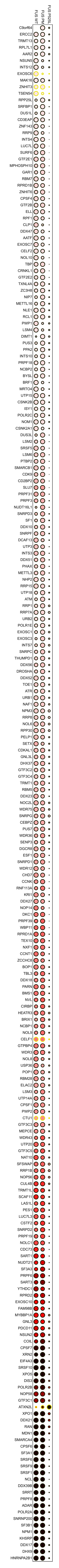

3K. CORUM:351 Spliceosome

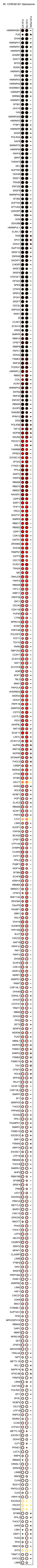

3L. GO:0006412 Translation

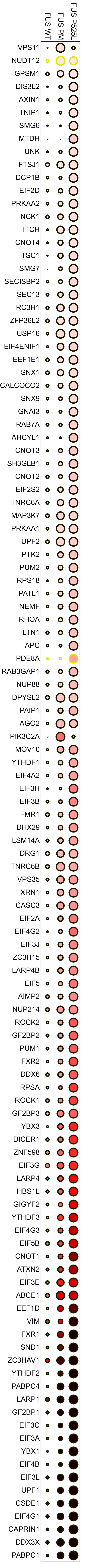

3M. GO:0050684 regulation of mRNA processing

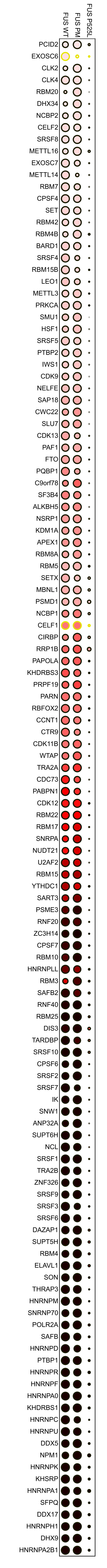

Supplement: Supporting Figure 3 [file mmc8.pdf]
