# Supplementary material for: Proximity-based labeling reveals DNA damage–induced phosphorylation of fused in sarcoma (FUS) causes distinct changes in the FUS protein interactome
Source: J Biol Chem. 2022 Jun 14;298(8):102135. doi: 10.1016/j.jbc.2022.102135 (PMC9372748; doi:10.1016/j.jbc.2022.102135)
Supplement: Supporting Figure 4 [file mmc9.pdf]

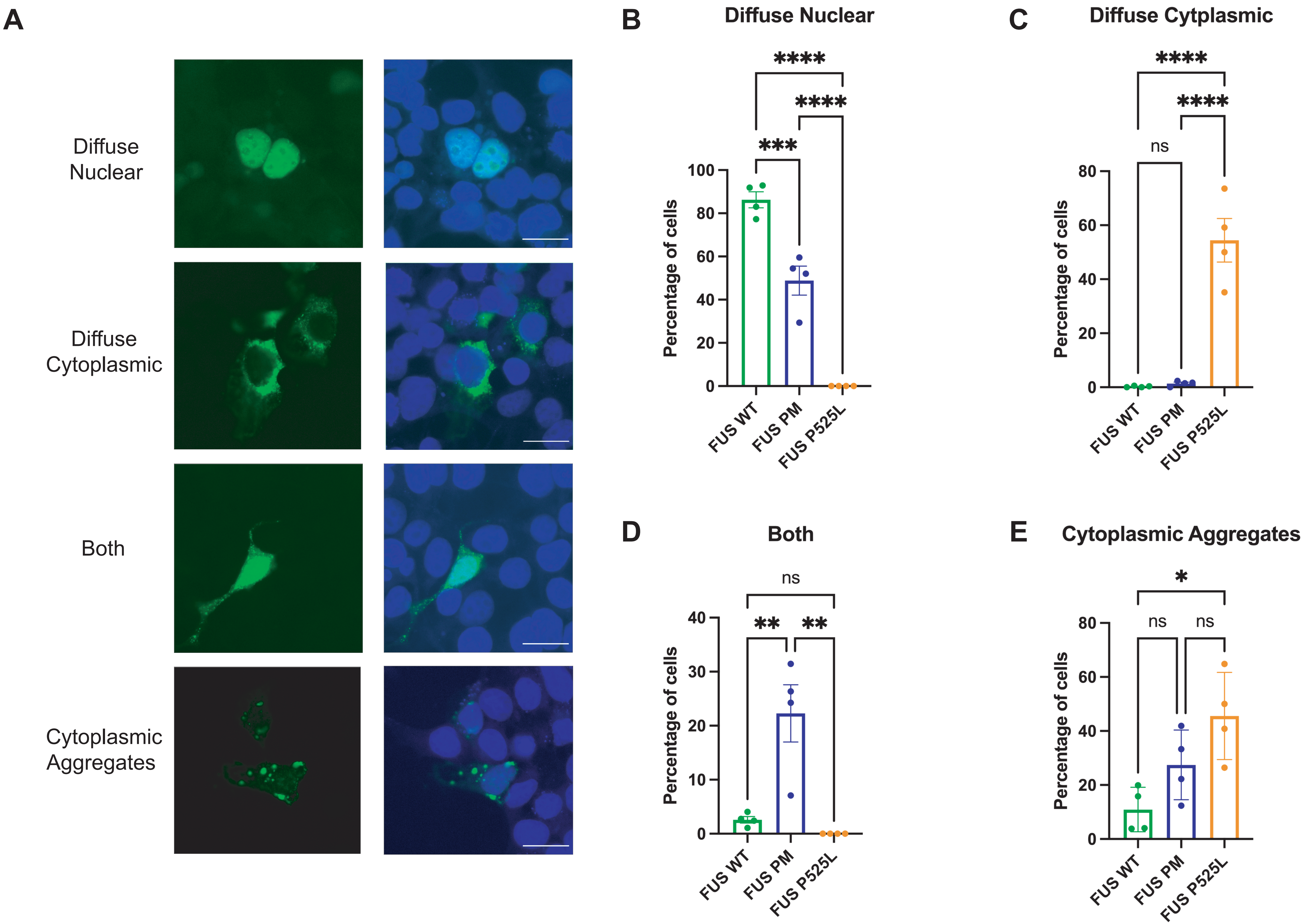

A
Diffuse Nuclear
Diffuse Cytoplasmic
Both
Cytoplasmic Aggregates
B
Diffuse Nuclear
****
****
***
Percentage of cells
100
80
60
40
20
0
FUS WT
FUS PM
FUS P525L
C
Diffuse Cytplasmic
****
****
ns
Percentage of cells
80
60
40
20
0
FUS WT
FUS PM
FUS P525L
D
Both
ns
**
**
Percentage of cells
40
30
20
10
0
FUS WT
FUS PM
FUS P525L
E
Cytoplasmic Aggregates
*
ns
ns
Percentage of cells
80
60
40
20
0
FUS WT
FUS PM
FUS P525L

Supplemental Figure 4. FUS PM is more cytoplasmic and forms more aggregates than FUS WT.

Cells were transfected with a GFP-Strep-tagged FUS variant (FUS WT, FUS PM, and FUS P525L). The same FUS variants were used in Figure 4. On average 165 cells were then classified into one of four categories (representative images of the classifications scheme used shown in (**A**)) for FUS localization: (**B**) Diffuse Nuclear (signal was spread diffusely throughout the nucleus), (**C**) Diffuse Cytoplasmic (signal was spread diffusely throughout the cytoplasm), (**D**) Both (signal was spread diffusely throughout the nucleus and cytoplasm) and (**E**) Cytoplasmic Aggregates (signal was present in cytoplasmic punctate). The boundaries of nucleus were determined using the DAPI signal. (**B-E**) The percentage of cells in each category was calculated for each group and a one-way ANOVA was performed to determined significance (n=4). Error bars indicate mean ± SEM. Scale bars represents 20 μm.
